# Metabolomics and proteomics insights into subacute ruminal acidosis etiology and inhibition of proliferation of yak rumen epithelial cells in vitro

**DOI:** 10.1186/s12864-024-10242-0

**Published:** 2024-04-22

**Authors:** JunMei Wang, Liyuan Shi, Xiaohong Zhang, Rui Hu, Ziqi Yue, Huawei Zou, Quanhui Peng, Yahui Jiang, Zhisheng Wang

**Affiliations:** Key Laboratory of Low Carbon Culture and Safety Production in Cattle in Sichuan, Animal Nutrition Institute, Sichuan Agricultural University, Chengdu, 611130 China

**Keywords:** Short-chain fatty acids, Low pH, LPS, Yak rumen epithelial cells, Metabolomics, Proteomics

## Abstract

**Background:**

Untargeted metabolomics and proteomics were employed to investigate the intracellular response of yak rumen epithelial cells (YRECs) to conditions mimicking subacute rumen acidosis (SARA) etiology, including exposure to short-chain fatty acids (SCFA), low pH5.5 (Acid), and lipopolysaccharide (LPS) exposure for 24 h.

**Results:**

These treatments significantly altered the cellular morphology of YRECs. Metabolomic analysis identified significant perturbations with SCFA, Acid and LPS treatment affecting 259, 245 and 196 metabolites (VIP > 1, *P* < 0.05, and fold change (FC) ≥ 1.5 or FC ≤ 0.667). Proteomic analysis revealed that treatment with SCFA, Acid, and LPS resulted in differential expression of 1251, 1396, and 242 proteins, respectively (FC ≥ 1.2 or ≤ 0.83, *P* < 0.05, FDR < 1%). Treatment with SCFA induced elevated levels of metabolites involved in purine metabolism, glutathione metabolism, and arginine biosynthesis, and dysregulated proteins associated with actin cytoskeleton organization and ribosome pathways. Furthermore, SCFA reduced the number, morphology, and functionality of mitochondria, leading to oxidative damage and inhibition of cell survival. Gene expression analysis revealed a decrease the genes expression of the cytoskeleton and cell cycle, while the genes expression associated with inflammation and autophagy increased (*P* < 0.05). Acid exposure altered metabolites related to purine metabolism, and affected proteins associated with complement and coagulation cascades and RNA degradation. Acid also leads to mitochondrial dysfunction, alterations in mitochondrial integrity, and reduced ATP generation. It also causes actin filaments to change from filamentous to punctate, affecting cellular cytoskeletal function, and increases inflammation-related molecules, indicating the promotion of inflammatory responses and cellular damage (*P* < 0.05). LPS treatment induced differential expression of proteins involved in the TNF signaling pathway and cytokine-cytokine receptor interaction, accompanied by alterations in metabolites associated with arachidonic acid metabolism and MAPK signaling (*P* < 0.05). The inflammatory response and activation of signaling pathways induced by LPS treatment were also confirmed through protein interaction network analysis. The integrated analysis reveals co-enrichment of proteins and metabolites in cellular signaling and metabolic pathways.

**Conclusions:**

In summary, this study contributes to a comprehensive understanding of the detrimental effects of SARA-associated factors on YRECs, elucidating their molecular mechanisms and providing potential therapeutic targets for mitigating SARA.

**Supplementary Information:**

The online version contains supplementary material available at 10.1186/s12864-024-10242-0.

## Introduction

There is increasing evidence of the crucial role the ruminal epithelium plays in.

nutrient absorption, metabolism, intracellular pH regulation, immunity, and the maintenance of the ruminal-host barrier, which are vital for ruminant health and productivity [1]. In ruminants, short-chain fatty acids (SCFA) are produced mainly by the rumen microbiome fermentation of cellulose, fibers, starch, and sugars [2, 3]. The use of high-concentrate diets to enhance short-term growth and fattening, results in excessive fermentation and elevated SCFA production [4–6]. Higher concentration of SCFA in the rumen fluid are associated with lower ruminal pH and the increased acid load causes SARA and damage or inflammation to rumen epithelium [7, 8]. Moreover, the concentration of lipopolysaccharide (LPS) in ruminal fluid rises due to the lysis of gram-negative bacteria during SARA, and LPS was discovered to translocate more readily across the ruminal wall, causing the pH decompensation-induced ruminal imbalance to worsen [9, 10]. Domestic and international researchers utilize pH values of 5.5, 5.6, or 5.8 as critical thresholds to assess SARA, with SARA occurrence and severity determined by the duration (310 min per day) and area under the curve of rumen pH persistently below these thresholds [11]. There are various opinions regarding the mechanisms of SARA, which primarily involve endotoxins (LPS), histamine, lactic acid, and organic acidosis theory [12, 13].

Yak is a distinctive breed of cattle that has evolved to survive in the harsh environment of the Qinghai-Tibet Plateau, which is located at elevations between 3000 and 5000 m above sea level [14]. Compared with other ruminants, yaks exhibit notable differences in gut function, characterized by a higher density of ruminal papillae and more pleats in the mucosa surface of their abomasum, as reported [15]. These adaptations increase the surface area of gastrointestinal tract and enhance absorptive capacity, enabling yaks to absorb energy more efficiently by up-regulating nutrient transporters and exhibit higher energy utilization and metabolic efficiency [16]. Due to the growing yak population and the extensive degradation of grasslands in recent years, cold-season supplemental feed or modern intensive feeding models have been implemented to enhance yak productivity [17–20]. Similarly, the practice of supplementary feeding or short-term housing for fattening yaks, with high-concentrate diets primarily based on grain starch as the main energy source, often leads to an accumulation of excessive SCFA in the rumen [21, 22]. Studies have also demonstrated that yaks exhibit higher SCFA production during ruminal fermentation compared to cattle at the same diet level or with the same substrate [23, 24]. These findings suggest a higher susceptibility of yaks to SARA.

Previous studies have demonstrated that the stratified squamous epithelium that makes up the ruminal epithelium encompasses the stratum corneum, stratum granulosum, stratum spinosum, and stratum basale [25, 26]. The intricate regulatory mechanisms involved in metabolic, physiological, and developmental processes across the various layers of epithelial cells are reflected in the unique structure of the ruminal epithelium. The stratum basale is enriched with mitochondria that generate adenosine triphosphate (ATP), providing energy for nutrient transport (VFAs, ketones, lactate, urea, minerals, etc.), tissue morphogenesis, pH regulation, and other systemic metabolic functions such as hormone and immune-related metabolism [27, 28]. However, the research on SARA etiology in YRECs remains limited, particularly regarding the molecular mechanisms involved. Proteomics and metabolomics comprehensively analyze changes in intracellular proteins and metabolites, enhancing data coverage and resolution to provide comprehensive information, aiding in the discovery of potential biomarkers and key pathways, thereby facilitating a deeper understanding of the complexity of injury mechanisms [29]. Therefore, this study employed metabolomics and proteomics techniques to elucidate the cellular-level toxic impact of high concentration of SCFA, low pH, and LPS on YRECs, shedding light on the pathogenesis of acidosis and providing novel insights for the future regulation of ruminal epithelial cell proliferation and health in yaks.

## Materials and methods

### Cell culture and treatment

We successfully established the yak rumen epithelial cell line (SV40T-YREC-hTERT) in our laboratory, which was then cultured within Roswell Park Memorial Institute-1640 (RPMI 1640, Gibco, USA) containing 10% fetal bovine serum (FBS, Gibco, USA), 100 U/mL streptomycin, and 100 U/mL penicillin at 37 °C in an incubator with 5% CO_2_ [30]. This cell line was established by isolating and culturing primary cells from rumen epithelial tissue obtained from adult yaks after electric shock, exsanguination and slaughter, and skinning according to standard commercial procedures. The yaks slaughtered in this study were not used anesthesia/euthanasia. The cell line was identified in the China Center for Type Culture Collection (CCTCC No. C2021245), and the cell line was free from species cross-contamination and mycoplasma contamination. To initiate subculture, the cells were exposed to a mixture comprising 0.25% trypsin and 0.02% EDTA for digestion, seeded into a 150 mm culture dish (Corning, NY, USA) overnight to 100% confluency for the following experiments. The cells were respectively treated with SCFA, low pH, and LPS with an equivalent volume of culture media for 24 h. The control group cells were treated with the same volume of RPMI-1640 medium instead (CONT). Notably, the concentration selection of the SCFA treatment group was based on our previous investigation of the cytotoxicity of SCFA on YRECs for 24 h. The concentration of 120 mM SCFA (6 mM sodium butyrate, 30 mM sodium propionate, and 84 mM sodium acetate; Sigma-Aldrich, MO, USA) inhibited cell viability by approximately 50% for 24 h as SCFA group (SCFA) [30]. We have adjusted medium pH 5.5 was acidified with HCl and treated cells for 24 h as acidic environment group (Aci) [31].The cytotoxic concentrations of LPS (From *Escherichia coli 055: B5*; Sigma-Aldrich, MO, USA) were determined by treating cells with different concentrations of LPS (5, 10, 20, 40, 80, 100, 120, 140 µg/mL) for 24 h. The concentration of 100 µg/mL LPS inhibited cell viability by approximately 10% for 24 h as LPS group (LPS).The cell viability was determined in accordance with the guidelines stipulated by the manufacturer of the cell counting kit-8 assay (CCK‐8; AbMole, Shanghai, China). In brief, the wells received 10 µL of the CCK-8 reaction solution, and 100 µL of the culture medium was retained. The absorbance was measured at 450 nm using a microplate reader after 2 h of incubation at 37 °C. The cell growth curve was produced, with the incubation period serving as the x-axis and the average optical density (OD) value as the y-axis.

### UHPLC-MS/MS-based investigations of non-targeted metabolomics

After the cells had been treated for 24 h, an 80% cooled methanol aqueous solution was introduced for 5 min to quench enzymatic reaction processes. Following cell harvest, a centrifugation step was performed at 5,000 rpm, 4 °C for 1 min. The freeze-drying process was applied to the supernatant, which was then dissolved with 10% methanol. The LC-MS/MS system analysis was then injected with the solution [32, 33]. The metabolic profiling was conducted on a Vanquish UHPLC system (Thermo Fisher, Germany) equipped with an Orbitrap Q ExactiveTM HF mass spectrometer (Thermo Fisher, Germany) in Novogene Co., Ltd. (Beijing, China) as previously reported [34]. Thereafter, peak alignment, peak selection, and quantification for each metabolite were performed using the UHPLC-MS/MS raw data loaded into the Compound Discoverer 3.1 (CD3.1, ThermoFisher) [35].

### Proteomic analysis

After 24 h of cell treatment with three biological replicates per treatment group, they were lysed in the cell-culture dish using a radioimmunoprecipitation assay (RIPA) lysis buffer (Beyotime, Shanghai, China) containing protease inhibitor (Beyotime, Shanghai, China) and 1 mM phenylmethanesulfonyl fluoride (PMSF). Subsequently, the Bradford protein quantification reagent (Beyotime, Shanghai, China) was used to determine the protein concentration. After 12% SDS-Page gel electrophoresis, the protein bands were stained with Coomassie bright blue R-250, and the protein quality was detected after decolorization until the bands were clear. The trypsin-digested peptides were tagged with Tandem Mass Tagging (TMT) Kits and Reagents (Thermo Fisher Scientific, Bremen, Germany) according to previous studies [36]. Briefly, protein samples were combined with DB protein dissolution solution (8 M urea, 100 mM TEAB, pH = 8.5) to a final volume of 100 µL. After adding trypsin and 100 mM TEAB buffer, the mixture was incubated at 37 °C for 4 h. Subsequently, additional trypsin and CaCl_2_ were introduced for overnight digestion. pH adjustment to less than 3 was performed using formic acid, followed by centrifugation at room temperature for 5 min at 12,000 g to obtain the supernatant. The TMT-labeled peptide mixture was fractionated on a Rigol L3000 HPLC using a C18 column (Waters BEH C18 4.6 × 250 mm, 5 μm). Subsequently, the EASY-nLC™ 1200 nanoliter UHPLC system and the homemade analytical column (25 cm×150 μm, 1.9 μm) were used for liquid chromatographic elution after the injection of 1 µg of supernatant of each fraction. The Orbitrap Exploris 480 was used in conjunction with a plus FAIMS mass spectrometer (Thermo Fisher Scientific, Bremen, Germany) to generate raw mass spectrometry data using a data-dependent acquisition mode [37].

### Quantitative real-time reverse transcription polymerase chain reaction analysis (qRT-PCR)

By following the guidelines of the manufacturer, the cells were processed using a Trizol reagent to extract the total ribonucleic acid (RNA) (Yeasen Biotechnology, China). 1 µg of total RNA was used to synthesize complementary DNA (cDNA) utilizing the Hifair™ II 1st Strand cDNA Synthesis Super Mix for qPCR kit (Yeasen Biotechnology, China), and PCR amplification was conducted using the Hieff UNICON® Universal Blue qPCR SYBR Green Master Mix kit (Yeasen Biotechnology, China). The relative amounts of the mRNAs were normalized with the housekeeping gene GAPDH for each sample. The 2^(− ΔΔCt)^ method was adopted to determine the fold change of mRNA expression [38]. Table S1 contains a list of the gene-specific primers utilized in the current investigation.

### Measurement of cell cycle

The cell suspensions of the samples were collected, fixed with ethanol at a 70% concentration on ice, and subsequently resuspended in 500 µL of a solution consisting of propidium iodide (Beyotime Biotechnology, China) and RNAase for staining [39]. Afterward, flow cytometry (BDVerse, BD, Germany) was used to analyze the cell suspensions. ModFit (Topsham, ME, USA) was used to evaluate the data.

### The content of filamentous actin (F-actin) was determined using rhodamine phalloidin

In brief, a PBS solution that contained 3.75% formaldehyde was used to fix the cell samples before being permeabilized at room temperature for 10 min with PBS solution with 0.5% Triton X-100. After incubating the samples with 5% bovine serum albumin (BSA), they were stained with 2U/mL rhodamine-labeled phalloidin binding to F-actin (Solarbio, China) [40]. The images were observed and collected under a fluorescence microscope (DMI 4000 B, Leica Microsystems Ltd).

### Specific fluorescent staining of mitochondria in living cells

When the cells were treated, each well received 500 nmol/L MitoTracker Green or Red (Beyotime Biotechnology, China) before incubation at 37 °C for half an hour. MitoTracker stain is preferentially absorbed by mitochondria with intact outer membrane potential and becomes green or red fluorescent reflecting viable mitochondria [41]. The images were observed and collected under a fluorescence microscope (DMI 4000 B, Leica Microsystems Ltd) and quantified by ImageJ analysis of the mean fluorescence intensity.

### Glutathione metabolism, oxidative stress-related enzyme activities, and inflammation-related cytokines were detected by kits

After cell samples were processed, cell deposition was collected and resuspended with 1 mL PBS in 1.5 mL centrifuge tubes, and then lysed in an ice bath using a probe sonicator for 20% power or 200 W, 3s ultrasonic, 10s intervals, repeated 30 times. After centrifuging the samples at 4 °C (12,000 rpm, 5 min), the resulting supernatant was obtained and utilized for enzyme-linked immunosorbent assay (ELISA, Sinobestbio, China) analysis and kits detection. Subsequently, ELISA was employed to determine the levels of tumor necrosis factor-α (TNF-α), matrix metalloproteinase-9 (MMP-9), and interleukin-1β (IL-1β), and intracellular cyclooxygenase 2 (COX-2) content. The detection of nicotinamide adenine dinucleotide phosphate oxidase (NADPH-OX), glutathione peroxidase (GSH-Px), hydrogen peroxide (H_2_O_2_), glutathione-s-transferase (GST), reactive oxygen species (ROS) and ATP were meticulously performed as per kit instructions (Bonoheng Biotechnology, China).

### Data processing and statistical analysis

For metabolomics data analysis in the metaX software, the widely used multivariate statistical techniques include principal component analysis (PCA) and orthogonal partial least-squares discriminant analysis (OPLS-DA) [42]. Selection of the possible differential metabolites was done using the criteria of VIP > 1, *P*-value < 0.05, and fold change (FC) ≥ 1.5 or FC ≤ 0.667. The identified metabolites were subjected to pathway analysis with MetaboAnalyst (http://mirror.metaboanalyst.ca/) and mapped into pathways as illustrated in the Kyoto Encyclopedia of Genes and Genomes (KEGG) (http://mirror.metaboanalyst.ca/). An enrichment was interpreted as statistically significant when *P* < 0.05.

Screening of raw data, quality control, and protein function annotation were the key components of proteomics data analyses. Each run-yielded spectra were searched separately against 1,062,060-UniProt-bos mutus-filtered-organism__Bos mutus (wild yak) [72,004] _. Fasta (38,178 sequences) database by the search engines: Proteome Discoverer 2.4 (PD 2.4, Thermo). Differentially expressed proteins (DEPs) were pairwise comparisons between two compared sample with FC ≥ 1.2 or ≤ 0.83 and a significance threshold of unadjusted with *t-test* for *P* < 0.05. The InterProScan program was employed to execute Gene Ontology (GO) and InterPro (IPR) functional analyses, against the non-redundant protein database, which includes PANTHER, ProSite, SMART, ProDom, PRINTS, and Pfam [43]. The differentially significant expression proteins of each comparison pair were annotated with subcellular localization information using the Cell-mPLOC 2.0 website (http://www.csbio.sjtu.edu.cn/bioinf/Cell-PLoc-2/), and protein transmembrane domain prediction was conducted using the TMHMM 2.0 online software. For the analysis of the DEP family and pathway, the Clusters of Orthologous Groups (COG) and KEGG databases were employed [44]. The Search Tool for retrieving the Interacting Genes/Proteins database(http://STRING.embl.de/) was employed as previously described [45] for the analysis of protein-protein interaction (PPI). Cytoscape was used to map the protein interaction network, employing the Degree topological analysis method from cytoHubba to analyze significantly different proteins, rank them based on scores, and filter key proteins based on high Degree scores.

The format of mean ± standard error (SEM) was utilized to display the data, and the data were analyzed using one-way analysis of variance (ANOVA), followed by subsequent Tukey’s multiple comparison test. The graphs were performed using Graph Pad Prism (v. 9.2.0). *P* < 0.05 was indicated as a statistical significance difference.

## Results

### The morphology of YRECs exposure by SCFA, low pH, and LPS

**Fig. 1:**
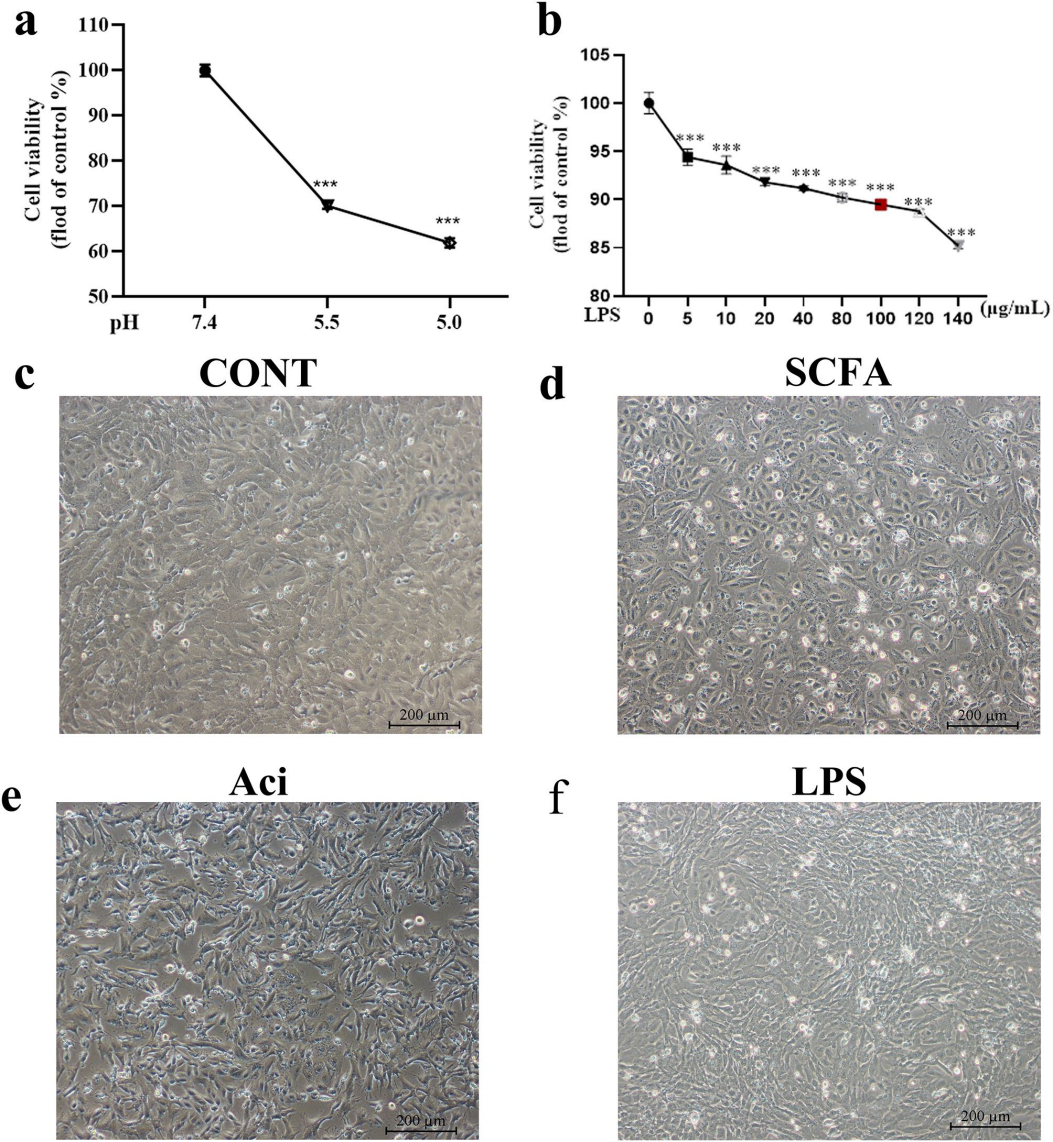
Effects of short-chain fatty acids (SCFA), low pH acidic environment (Aci), and lipopolysaccharides (LPS) on cell morphology and cell viability of YRECs for 24 h. The effect of different pH levels (pH5.5 and pH5.0) on cell viability for 24 h. Cells were treated with the indicated concentrations (5, 10, 20, 40, 80, 100, 120, 140 µg/mL) of LPS for 24 h. The results were presented as the percent cell viability compared with the untreated control group (**b**). Data are the means ± SEM, *n* = 3; ***, *P* < 0.001 compared with the control group. Morphological characterization of damage to YRECs after exposure to SCFA (**d**), Aci (**e**), and LPS (**f**) respectively (100x)

The cellular morphology was significantly altered after treatment with different factors associated with SARA etiology for 24 h. Notably, Low pH acidic environment significantly inhibited cell viability (*P* < 0.001; Fig. 1a). The impact of LPS on cell viability exhibited a concentration-dependent effect (*P* < 0.001; Fig. 1b). We selected LPS with low pH5.5, which inhibited cell viability by about 30%, and LPS inhibited cell viability by about 10% to further study the effects of acidic environment and LPS on cell proteins and metabolites. As shown in Fig. 1c, the control cells were tightly adhered to the cell culture bottle, displaying irregular morphology, well-defined stereoscopic features, and favorable refractive properties under the ordinary light microscope. In contrast, the SCFA-treated group displayed floating dead cells with reduced cell refractivity, diminished stereoscopic appearance, cell shrinkage, widened intercellular gaps, and a notable decrease in cell numbers (Fig. 1d). With low pH the morphology of the epithelium cells was destroyed, the number of cell deaths higher and the gap between cells appeared larger (Fig. 1e). Although the cells look confluent like the control cells, intercellular gap width and cytoplasmic enlargement significantly increased in the LPS-treated group at a concentration of 100 µg/mL (Fig. 1f).

### Differential metabolite analysis

**Fig. 2:**
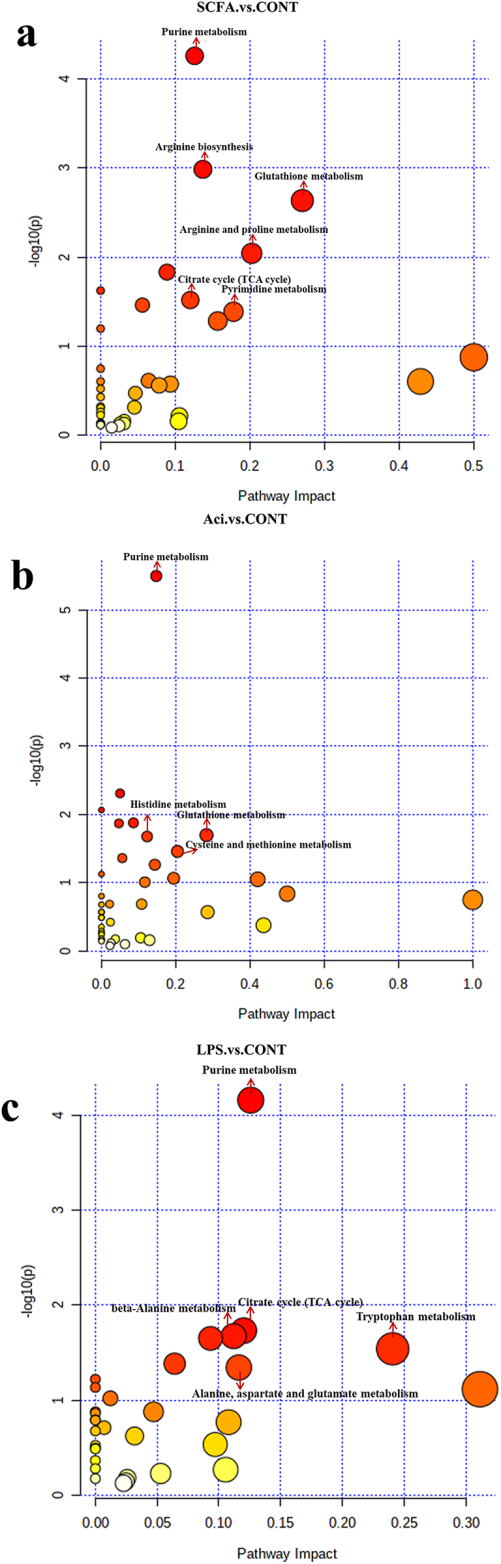
Differential metabolites of SCFA, Aci, LPS, and CONT with six replicate samples per group in YRECs. As per the detected differential metabolites, MetaboAnalyst 5.0 was utilized to examine the probable metabolic pathways. (**a**) SCFA vs. CONT. (**b**) Aci vs. CONT. (**c**) LPS vs. CONT

After data quality control of metabolomics was qualified (Fig. S1a-d), A satisfactory separation between the CONT and treatments could be seen in the PCA and OPLS-DA score plots (Fig. S2a, b). Under the condition of FC > 1.5 or FC < 0.667, *P* < 0.05 and VIP > 1, metabolites were sorted from highest to lowest according to the VIP score, high concentrations of SCFA significantly affected 259 metabolites (Table S2), 6beta-Hydroxytestosterone, (+/-)12(13)-DiHOME, 4-butylresorcinol, gamma-glutamyltyrosine and 5-oxoETE were among the metabolites with elevated levels, and glutathione, sedoheptulose1,7-bisphosphate, L-glutathione(reduced), L-ornithine and L-arginine had reduced levels (*P* < 0.001) (Table S3.1). Various metabolites were used to study metabolic pathways (Impact > 0.1, *P* < 0.01). As shown by the KEGG pathway enrichment, six functional pathways were significantly enriched in high-concentration SCFA, including purine metabolism, glutathione metabolism and arginine biosynthesis (Fig. 2a).

A high concentration of SCFA will reduce pH value in rumen fluid, and further test the effect of acidic environment on rumen epithelial cells. Under the condition of FC > 1.5 or FC < 0.667, *P* < 0.05 and VIP > 1, metabolites were sorted from highest to lowest according to the VIP score, the acidic environment significantly affected 245 metabolites (Table S2). 5-oxoproline, α -Lapachone, Hexadecanedioic acid, Deoxyinosine and 4-Oxoproline in the elevated metabolites and Pyridoxal 5-phosphate monohydrate, Hypotaurine, (+)-alpha-lipoic acid and deoxyadenosine in the metabolites with reduced levels (*P* < 0.001) (Table S4.1). Purine metabolism pathway affected by the acidic environment were significantly enriched (Impact > 0.1, *P* < 0.01) (Fig. 2b).

Addition of LPS to the culture medium significantly affected 196 metabolites (FC > 1.5 or FC < 0.667, *P* < 0.05 and VIP > 1) (Table S2), and (5-L-Glutamyl)-L-Amino Acid, Gamma-Glu-Leu, Ala-Leu, (2R)-2,3-dihydroxypropanoic acid and 8-iso-15-keto Prostaglandin E2 in the elevated metabolites and 2’- deoxyadenosine 5’-monophosphate (dAMP), dCMP, Thymidine 5’-monophosphate, L-Pipecolate.

,4-Guanidinobutyric acid, dTMP in the metabolites with reduced levels (*P* < 0.001) (Table S5.1). Purine metabolism pathway affected by LPS were significantly enriched (Impact > 0.1, *P* < 0.01) (Fig. 2c).

### Detection of DEPs by TMT-based proteome profiling of YRECs response to high concentration of SCFA

**Fig. 3:**
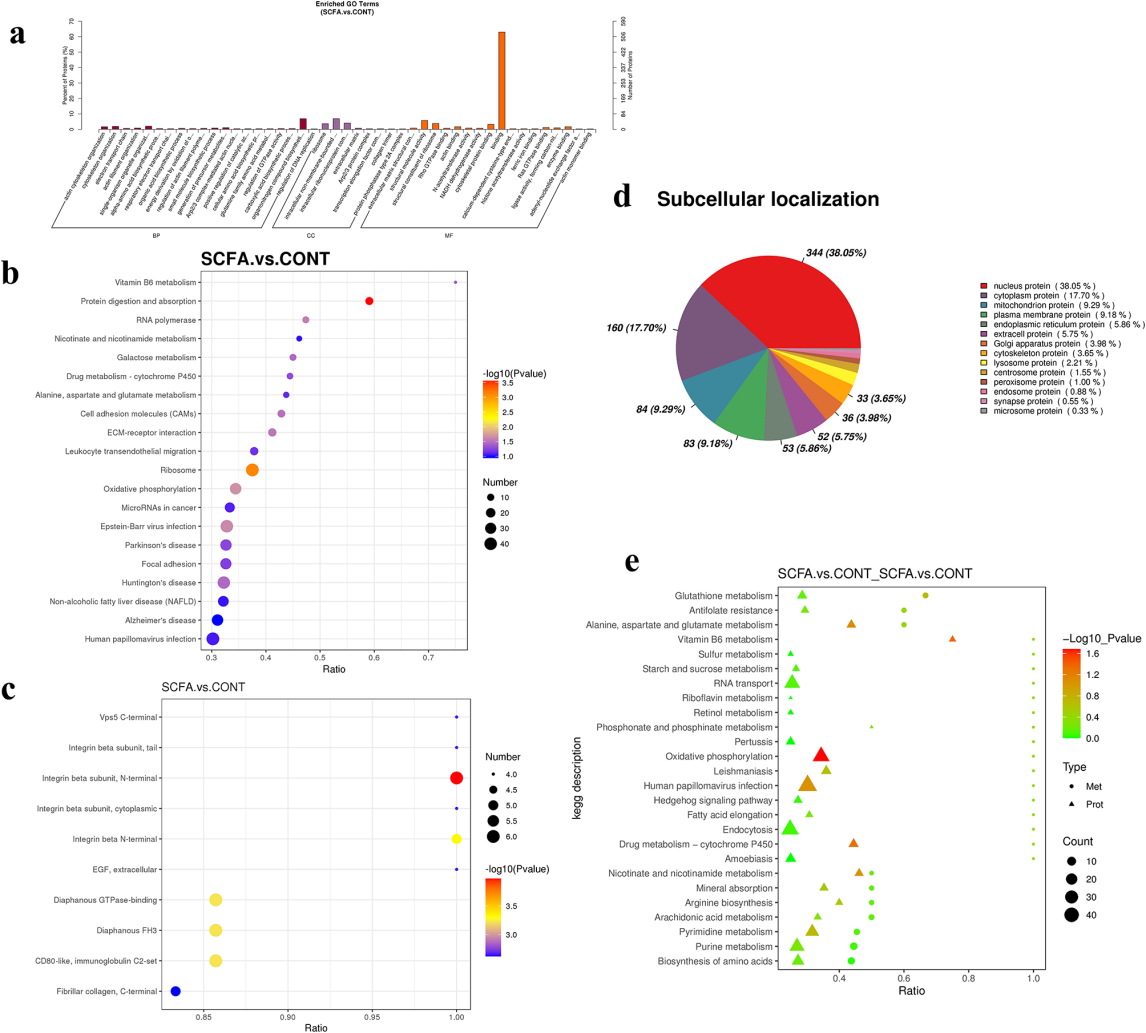
Differentially abundant proteins of SCFA and CONT by TMT-based quantitative proteome analysis in YRECs(*n* = 3). (**a**) Differentially abundant proteins in two comparison groups were classified according to molecular functions (MF), cellular components (CC), and biological processes (BP) using the enriched GO terms. The graphic displays the enrichment outcomes in three categories with a maximum of 20 in each (*P*-value ≤ 0.05). (**b**) KEGG pathway enrichment of differentially abundant proteins. The hypergeometric test’s P-value is indicated by the dot color. The differential protein count in the corresponding pathway is denoted by the size of the dots. (**c**) Structural domain enrichment bubble diagram of differentially abundant proteins. (**d**) The evaluation of differentially abundant proteins’ subcellular location. Integrative multi-omic data analysis revealed metabolites and proteins that could be linked to high concentration of SCFA-induced injury in YRECs. (**e**) The bubble map shows the enrichment of DEMs and DEPs in the same KEGG pathways. The horizontal coordinate is the ratio of differential metabolites or proteins improved in the pathway to the number of metabolites or proteins annotated in the pathway (Ratio). The metabolome-proteome co-enriched to the KEGG pathway is represented along the vertical coordinate

After data quality control (Fig. S1e-h), quantitative protein PCA scatter plots and the cumulative plot curves of coefficient of variance (CV) values of all proteins indicated significant differences between treatment groups identified by proteomics, and the samples have small CVs and good reproducibility (Fig. S2c, d). The SCFA-treated group expressed 1251 proteins differentially as compared to the CONT, including 698 proteins had higher abundance (FC > 1.2; *P* < 0.05), 553 proteins had lower abundance (FC < 0.83; *P* < 0.05) (Table S3.2). To further examine the functional features of the differently expressed proteins, the protein GO database annotated and enriched the selected differentially abundant proteins. Differentially expressed abundant proteins were classified and displayed into three ontological categories of biological process (BP), cellular component (CC), and molecular function (MF). SCFA-treated groups significantly enriched in 28 terms in BP, which are actin cytoskeleton organization, cytoskeleton organization, cytoskeleton organization, electron transport chain. The 8 terms were significantly enriched in CC, such as ribosome, intracellular non-membrane-bounded organelle, intracellular ribonucleoprotein complex. Seventeen terms were significantly enriched during MF and extracellular matrix structural constituent, structural molecule activity, structural constituent of ribosome (*P* < 0.05; Fig. 3a). A bubble plot of the KEGG pathway enriched for differential proteins was shown in Fig. 3b. The SCFA-treated group significantly affected 10 signal transduction pathways in contrast to the CONT (*P* < 0.05). Differential protein enrichment was highest in the vitamin B6 metabolism pathway, and upregulated proteins were primarily enriched in the ribosome pathway. High concentration of SCFA increased abundance of proteins involved in alanine, aspartate, glutamate metabolism, galactose metabolism, proteasome, arginine biosynthesis, RNA polymerase, and RNA transport (Fig. S3a). Lower abundance of proteins enriched for protein digestion and absorption pathway was statistically significant. High concentration of SCFA also inhibited proteins in pathways of oxidative phosphorylation, ECM-receptor interaction, regulation of actin cytoskeleton, focal adhesion, protein digestion, and absorption, Wnt signaling pathway, tight junction, and cell cycle (Fig. S3b). The findings of the differential protein-related structural domain enrichment indicated that the effects of integrin beta, vps5 C-terminal, EGF (epidermal growth factor; extracellular), and diaphanous GTPase-binding were significant (*P* < 0.05; Fig. 3c). Additionally, the subcellular localization data showed that nuclear proteins constituted 38.05% of the SCFA treatment group, while cytoplasmic proteins constituted 17.70%, mitochondrion protein constituted 9.29% and plasma membrane proteins constituted 9.18% (Fig. 3d). The proportion of transmembrane proteins with the predicted transmembrane helical structure with at least one transmembrane domain (TMHMM) in the differentially expressed proteins of SCFA-treated YRECs was 16.3% (Table S3.2). The StringDB protein interaction database (http://string-db.org/) was searched to identify differentially enriched proteins by PPI network interaction analysis. The interaction information between target proteins was obtained. To further understand the protein interactions between biological processes involved in the toxic injury of high concentration of SCFA to YRECs. In the PPI network, 542 proteins were found to interact with one or more proteins after removing undefined proteins in the SCFA treatment group (Fig. S4a). The top five interacting proteins were 40 S ribosomal protein S27a, glyceraldehyde-3-phosphate dehydrogenase, 60 S ribosomal protein L40, heat shock 70 kDa protein 1B, and 78 kDa glucose-regulated protein.

### Integrated analysis of metabolomics and proteomics of high concentration of SCFA on YRECs

KEGG pathway analysis was performed on the combined metabolomic and proteomic data to gain a comprehensive understanding of the mechanisms underlying the injury damage at high concentration of SCFA cause in YRECs. The findings illustrated the co-enrichment of the DEPs and differentially expressed metabolomics (DEMs) in 26 pathways (Fig. 3e; Table S3.3). The altered proteins and metabolites were mainly involved in the metabolism of nucleotides, glutathione, glutamate, aspartate, arachidonic acid, alanine, nicotinamide, and nicotinate as well as the biosynthesis of amino acids and arginine, RNA transport, and oxidative phosphorylation. Strong relationships between the differential proteins and metabolites were found by correlation analysis. Among them, the D-galactosamine interact with the protein NADH dehydrogenase [ubiquinone] 1 alpha subcomplex assembly factor 3 was highly positive correlated respectively (*r* = 0.99, *P* < 0.001) (Table S3.4).

### Functional damage of YRECs induced by the high concentration of SCFA

**Fig. 4:**
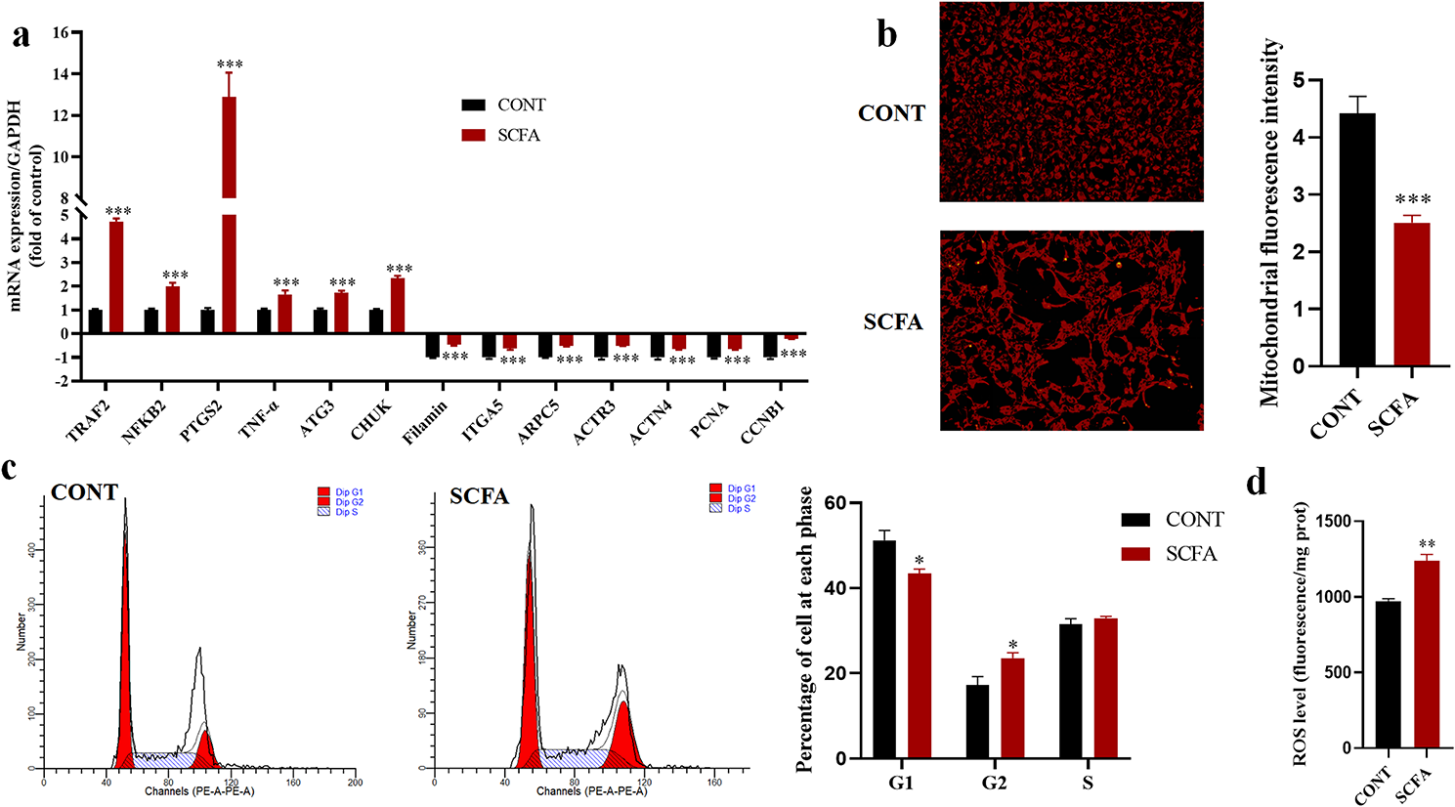
Cytotoxic damage induced by high concentration of SCFA exposure to YRECs. (**a**) Effects of high concentration SCFA exposure YRECs on the mRNA expression levels of filamin, ITGA5, ARPC5, ACTR3, ACTN4, PCNA, CCNB1, NFKB2, TRAF2, CHUK, PTGS2, ATG3, and TNF-α for 24 h. (**b**) The morphology and fluorescence intensity of mitochondrial staining in SCFA exposure YRECs. (Magnification 100×; red: Mitochondria). (**c**) Detection of cell cycle process in cells by flow cytometry and proportion of cells in the G0/G1, G2/M, and S phases. (**d**) Detection of ROS levels in cells. The format of mean ± SEM (*n* = 3) was used to present the data. *, ** and *** demonstrate significant deviations from the control at *P* < 0.05, *P* < 0.01 and *P* < 0.001 respectively

In this experiment, the real-time qPCR was used to verify the relevant cytotoxic damage at the transcriptional level. It was found that the expression of cytoskeleton and cell cycle-related genes (Filamin, ITGA5, ARPC5, ACTR3, ACTN4, PCNA, and CCNB1) were decreased. The expression of inflammation and autophagy-related genes (NFKB2, TRAF2, CHUK, PTGS2, TNF-α, and ATG3) was increased in YRECs exposed to high concentration of SCFA (*P* < 0.001; Fig. 4a). We used Mitotracker RedCMXRos to assess the alterations in mitochondrial morphology/distribution by fluorescence microscope. In contrast to the CONT, high concentration of SCFA reduced the number, morphology, and fluorescence intensity of mitochondria in YRECs, representing mitochondrial dysfunction (*P* < 0.001; Fig. 4b). In addition, we also detected cell cycle changes by flow cytometry, which showed G2 phase arrest, and the expression of G2-related proteins was inhibited by omics assay (*P* < 0.05; Fig. 4c). These results again indicated that high concentration of SCFA inhibited cell survival. Mitochondria are also the leading site of intracellular ROS generation. A high concentration of SCFA induced a high content of ROS in cells, resulting in oxidative damage (*P* < 0.01; Fig. 4d).

### TMT-based proteome analysis of the impact of low pH acidic environment on YRECs to identify DEPs

**Fig. 5:**
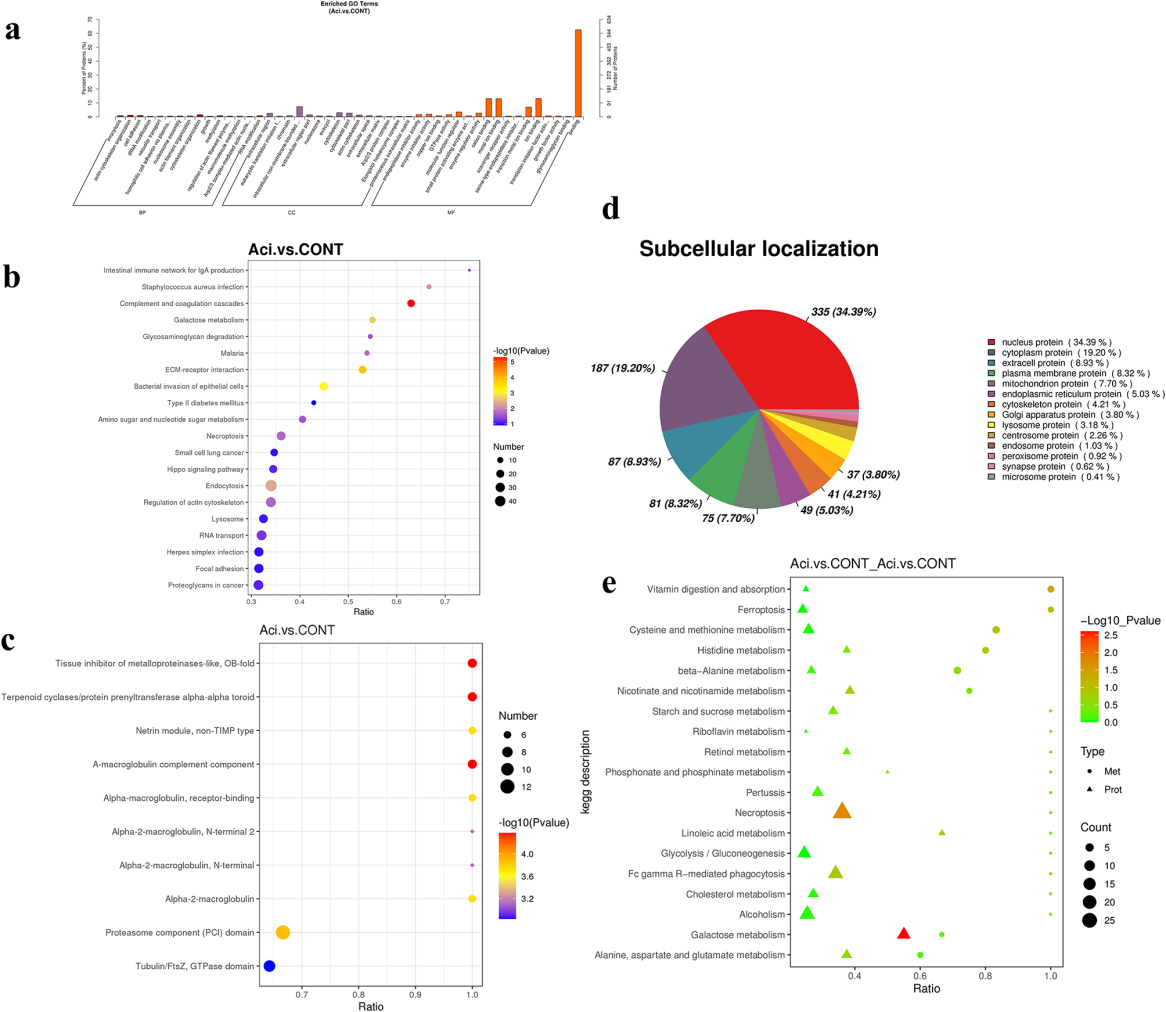
Differentially abundant proteins of Aci and CONT by TMT-based quantitative proteome analysis in YRECs(*n* = 3). (**a**) Differentially abundant proteins in two comparison groups were classified according to molecular functions (MF), cellular components (CC), and biological processes (BP) using the enriched GO terms. The figure shows the enrichment results in three categories, up to 20 of each (P-value ≤ 0.05), and the percentage of the vertical coordinate represents x/n in the table. (**b**) Proteins with differential levels of abundance are enriched in KEGG pathways. The P-value from the hypergeometric test is depicted through the color variation of each dot in the graph. Each dot’s size corresponds to the number of differential proteins identified within the corresponding pathway. (**c**) Structural domain enrichment bubble diagram of differentially abundant proteins. (**d**) Subcellular localization analysis of differentially abundant proteins. Integrative multi-omic data analysis revealed metabolites and proteins that may be associated with low pH acidic environment-induced injury in YRECs. (**e**) The bubble map shows the enrichment of DEMs and DEPs in the same KEGG pathways. The ratio of differential metabolites or proteins to the total number of metabolites or proteins in the pathway is depicted on the horizontal axis (Ratio). On the vertical axis, the value reflects the extent of co-enrichment between the metabolome and proteome within the KEGG pathway

Compared with the CONT, the low pH acidic environment-treated group differentially expressed 1396 proteins, including 675 proteins had higher abundance (FC > 1.2; *P* < 0.05), 721 proteins had lower abundance (FC < 0.83; *P* < 0.05) (Table S4.2). Low pH acidic environment treated group differentially expressed proteins significantly enriched in 15 terms during BP, including exocytosis, actin cytoskeleton organization, cell adhesion. The 15 terms were significantly enriched in CC, such as extracellular region, eukaryotic translation initiation factor 3 complex, chromatin. The 17 terms were significantly enriched in MF, and comprised endopeptidase inhibitor activity, copper ion binding, GTPase activity (*P* < 0.05; Fig. 5a). A bubble plot of the KEGG pathway enriched for differential proteins was shown in Fig. 5b. The low pH acidic environment-treated group significantly affected 12 signal transduction pathways as opposed to the CONT (*P* < 0.05). Differential protein enrichment was highest in the intestinal immune network for the IgA production pathway. The upregulated expression of proteins enriched to differential protein in the complement and coagulation cascades pathway was the most reliable and statistically significant (*P* < 0.001; Fig. S3c). Low pH acidic environment also activated galactose, nucleotide sugar, and amino sugar metabolism, as well as RNA degradation, and ECM-receptor interaction were enriched for these pathways. Low pH acidic environment also inhibited protein abundance of the actin cytoskeleton, oxidative phosphorylation, mTOR signaling pathway, Fc gamma R-mediated phagocytosis, and Toll-like receptor signaling pathway (Fig. S3d).

Low-pH treatment significantly increased the effects of terpenoid cyclases/protein prenyltransferase alpha-alpha toroid, tissue inhibitor of metalloproteinases-like (OB-fold), a-macroglobulin complement component, and proteasome component (PCI) domain, according to the structural domain enrichment findings of the differential proteins (*P* < 0.05; Fig. 5c). Furthermore, the subcellular localization data revealed that nuclear proteins accounted for 34.39% of the differentially abundant proteins in the low pH acidic environment treatment group, with cytoplasm、extracell and plasma membrane proteins accounting for 19.2%、8.93% and 8.32%, respectively (Fig. 5d). The proportion of transmembrane proteins with the predicted transmembrane helical structure with at least one TMHMM in the differentially expressed proteins of low pH -treated YRECs was 15% (Table S4.2). The 741 proteins were found to interact with one or more proteins in the low-pH treatment group (Fig. S4b). The top five interacting proteins were UV excision repair protein RAD23, glyceraldehyde-3-phosphate dehydrogenase, 78 kDa glucose-regulated protein, heat shock protein HSP 90-beta, and cell division protein kinase 1.

### Integrated analysis of metabolomics and proteomics of low pH acidic environment on YRECs

KEGG pathway analysis revealed co-enrichments for 26 pathways between DEMs and DEPs (Fig. 5e). The altered proteins and metabolites were mainly involved in methionine and cysteine metabolism, beta-alanine metabolism, vitamin digestion and absorption, histidine metabolism, ferroptosis, glutamate, aspartate, and alanine metabolism, as well as nicotinamide and nicotinate metabolism (Table S4.3). The results of correlation analysis revealed strong correlations between the differential proteins and metabolites. Among them, the metabolite (±)11(12)-EET interact with Ankyrin_rpt-contain_dom domain-containing protein was highly negative correlated (*r*=-0.99, *P* < 0.001) (Table S4.4).

### Functional damage of YRECs induced by low pH acidic environment

**Fig. 6:**
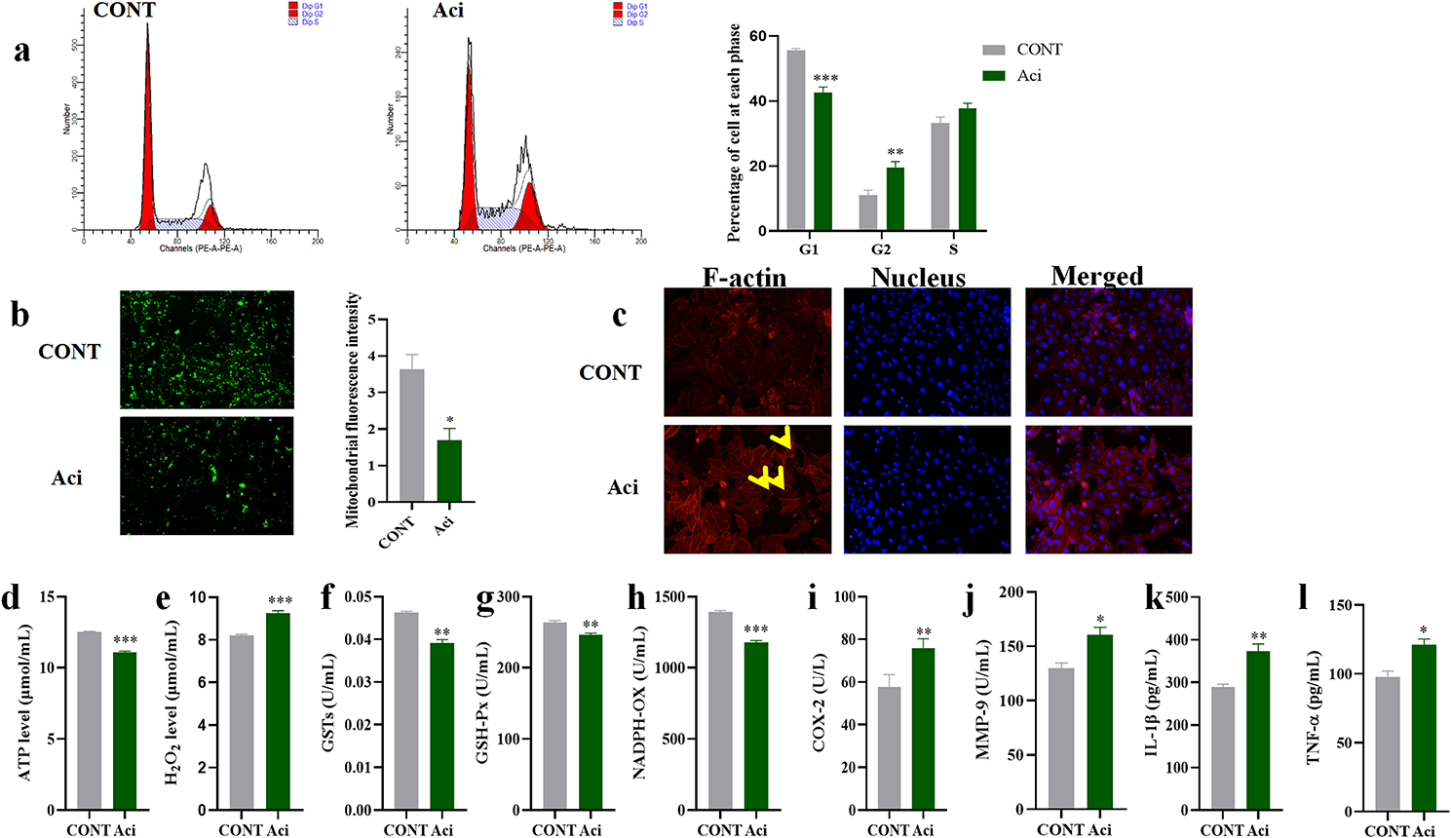
Cytotoxic damage induced by Aci exposure to YRECs. (**a**) The proportion of cells in the G0/G1, G2/M, and S phases as determined by flow cytometry. (**b**) The morphology and fluorescence intensity of mitochondrial staining in cells. (Magnification 100×; green: Mitochondria). (**c**) Phalloidin staining was used to determine the structure of F-actin (magnification 200×; red: F-actin). Effect of Aci exposure YRECs on intracellular ATP (d), H_2_O_2_ (**e**), GSTs (**f**), GSH-Px (**g**), NADPH-OX (**h**), COX-2 (**i**), MMP-9 (**j**), IL-1β (**k**) and TNF-α(**l**) expression levels. The format of mean ± SEM (*n* = 3) was used to present the data. *, ** and *** demonstrate significant variations from the control at *p* < 0.05, *p* < 0.01 and *p* < 0.001 respectively

The impact of an acidic environment on the cell cycle of YRECs was determined via flow cytometry. The findings demonstrated that the cell cycle was disordered and the G2 phase was arrested, both of which inhibited cell proliferation (*P* < 0.01; Fig. 6a). The YRECs were exposed to a low pH acidic environment before being stained with MitoTracker Green to evaluate mitochondrial integrity. The results showed that the fluorescence intensity and the mitochondrial activity decreased, and altered mitochondrial function and integrity (*P* < 0.05; Fig. 6b). Filamentous actin was visualized using phalloidin staining, thereby demonstrating the distribution of filamentous actin in the cells (Fig. 6c). The control group cells had an extensive cortical actin network. However, the low pH acidic environment changed the actin filaments from filamentous to punctate, which led to the rearrangement of actin filaments and the destruction of the cytoskeletal function of epithelial cells. The disruption of mitochondrial function was followed by a rise in the content of hydrogen peroxide and a reduction in intracellular ATP generation (*P* < 0.001; Fig. 6d, e), again demonstrating disruption of intracellular energy metabolism. At the same time, low pH inhibited the activities of metabolic enzymes related to glutathione metabolism (GSTs, GSH-Px, and NADPH-OX), thereby destroying the redox state in cells and inducing oxidative damage (*P* < 0.01, *P* < 0.01, *P* < 0.001; Fig. 6f-h). Furthermore, we examined the inflammation-related expressions of IL-1β, COX-2, MMP-9, and TNF-α. We found that low pH acidic environment significantly increased the inflammation-related molecules which suggests an increase in inflammatory response in the YRECs, leading to cell damage (*P* < 0.01, *P* < 0.05, *P* < 0.01, *P* < 0.05; Fig. 6i-l).

### TMT-based proteomic analysis of the impact of LPS on YRECs to identify DEPs

**Fig. 7:**
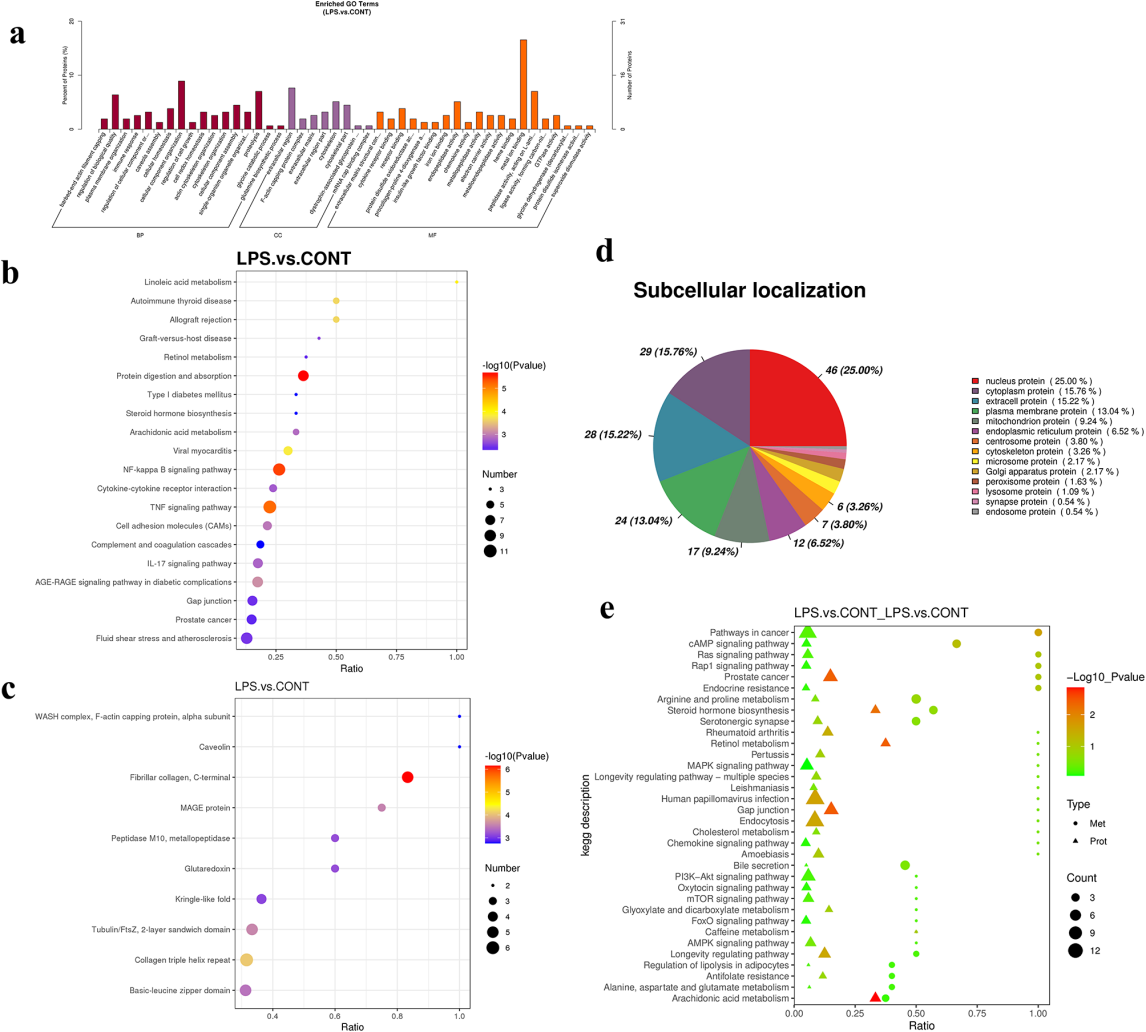
Differentially abundant proteins of LPS and CONT by TMT-based quantitative proteome analysis in YRECs(*n* = 3). (**a**) Differentially abundant proteins in two comparison groups were classified according to molecular functions (MF), cellular components (CC), and biological processes (BP) using the enriched GO terms. The figure shows the enrichment results in three categories, up to 20 of each (*P*-value ≤ 0.05). (**b**) Differentially abundant proteins enriched in KEGG pathways. On the horizontal axis, the value represents the proportion of differential proteins in a specific pathway relative to the total count of proteins detected in that pathway. The P-value from the hypergeometric test is depicted through the color variation of each dot in the graph. Each dot’s size corresponds to the number of differential proteins identified within the specific pathway. (**c**) Structural domain enrichment bubble diagram of proteins with differential abundance. (**d**) Subcellular localization analysis of differentially abundant proteins. Integrative multi-omic data analysis revealed metabolites and proteins that may be associated with LPS induced injury in YRECs. (**e**) The bubble map shows the enrichment of DEMs and DEPs in the same KEGG pathways. The ratio of differential metabolites or proteins to the total number of metabolites or proteins in the pathway is depicted on the horizontal axis (Ratio). On the vertical axis, the value reflects the extent of co-enrichment between the metabolome and proteome within the KEGG pathway

In contrast to the CONT, the LPS-treated group differentially expressed 242 proteins, including 117 proteins had higher abundance (FC > 1.2; *P* < 0.05), 125 proteins had lower abundance (FC < 0.83; *P* < 0.05) (Table S5.2). LPS-treated group significantly enriched in 17 terms in BP, which was barbed-end actin filament capping, regulation of biological quality and plasma membrane organization on differentially expressed proteins. The 8 terms were significantly enriched in CC, such as extracellular region, F-actin capping protein complex, extracellular matrix. The 24 terms were significantly enriched in MF and consisted of the extracellular matrix structural constitute, cytokine receptor binding, receptor binding (*P* < 0.05; Fig. 7a). A bubble plot of the KEGG pathway enriched for differential proteins was shown in Fig. 7b. The 39 signal transduction pathways were significantly affected by the LPS treatment relative to the CONT (*P* < 0.05). The linoleic acid metabolism pathway was highest enriched in DEPs. The upregulated expression of proteins enriched to DEPs in the TNF signaling pathway was the most reliable of the test and statistically significant (*P* < 0.001). LPS also activated the intestinal immune network for IgA production, IL-17 signaling pathway, cytokine-cytokine receptor interaction, and NF-kappa B signaling pathway (Fig. S3e). LPS also inhibited protein enriched for protein digestion and absorption, gap junction, phagosome, and endocytosis (Fig. S3f).

The structural domain enrichment results of the DEPs indicated that the effects of collagen triple helix repeat, fibrillar collagen (C-terminal), tubulin/FtsZ (2-layer sandwich domain), and MAGE protein were significant (Fig. 7c). Additionally, the subcellular localization data showed that nuclear proteins accounted for 25.00% of the differentially abundant proteins in the LPS treatment group, with cytoplasmic、extracell and plasma membrane proteins accounting for 15.76% 、15.22% and 13.04%, respectively (Fig. 7d). The proportion of transmembrane proteins with the predicted transmembrane helical structure with at least one TMHMM in the differentially expressed proteins of LPS-treated YRECs was 15% (Table S5.2). The top five differentially expressed proteins with the most interacting proteins in the LPS-treated group were tyrosine-protein kinase, cadherin-1, fibroblast growth factor receptor, nuclear factor NF-kappa-B p105 subunit, 72 kDa gelatinase (Fig. S4c).

### Integrated analysis of metabolomics and proteomics of LPS on YRECs

The co-enrichment of DEMs and DEPs was found in 34 pathways as demonstrated by the KEGG pathway analysis (Fig. 7e). The altered proteins and metabolites were primarily involved in multiple pathways, which include the cAMP signaling pathway, arachidonic acid metabolism, endocrine resistance, steroid hormone biosynthesis, serotonergic synapse, longevity regulating pathway, MAPK signaling pathway, gap junction (Table S5.3). The results of correlation analysis revealed strong correlations between the differential proteins and metabolites. Among them, the metabolite GDP interact with the protein YTH domain family protein 1, was highly positive correlated (r^2^ = 0.99, *P* < 0.001) (Table S5.4).

## Discussion

Maintaining a stable intraruminal environment is crucial for optimal gastrointestinal function and productivity in ruminants. Numerous factors have been reported to influence ruminal epithelial function, encompassing ruminal microecological factors and the physiological conditions of the host. Ruminal pH, molar concentrations of SCFA, and LPS serve as crucial indicators of a stable ruminal environment, reflecting the health status of ruminal fermentation [46–48]. SCFA plays a vital role in shaping the internal rumen environment, maintaining electrolyte balance, and serving as an energy source for host cells [49–51]. The concentration of SCFA in the rumen fluid ranges from around 60 mmol/L under normal diets to over 70 mmol/L in high-concentrate diets [22, 52]. Moreover, our previous experiment in vitro showed that the concentrations of SCFA above 60 mmol/L gradually and dosage-dependently suppressed the proliferation of YRECs, with an inhibition rate reaching approximately 50% at 120 mmol/L SCFA [30]. Ruminal pH is a crucial determinant of nutrient availability for absorption [53]. Nevertheless, previous studies have shown that luminal factor pH regulates cell proliferation and increases senescence, sloughing, or both in the rumen of goats [54]. Low ruminal pH, along with an elevated concentration of SCFA in the ruminal fluid and increased osmolality, has been reported to disrupt epithelial barrier function, thereby increasing the permeability of the ruminal epithelium, and result in the translocation of ruminal LPS, bacteria, or other microbe-associated molecular patterns (MAMP) molecules into peripheral tissue, inducing a systemic inflammatory response [55]. Additionally, previous findings indirectly indicate that different cell types exhibit varying tolerances to LPS [56, 57]. The damage caused by these factors that disturb the internal rumen environment should not be overlooked in YRECs. In the present study, morphological observations demonstrated hindered proliferation of YRECs and impaired cell morphology.

Metabolites are involved in enzyme-catalyzed chemical reactions that underlie cellular function, playing a crucial role in maintaining cellular homeostasis [58]. Metabolomics can detect multiple metabolites in critical metabolic pathways, which can help to deepen the understanding of the mechanisms behind metabolic disorders [59, 60]. In this study, metabolomics analysis revealed that excessive concentrations of SCFA, low pH acidic environment, and LPS significantly affected purine metabolic pathways. Purine metabolites, including adenine and guanine, are essential components of DNA, RNA, ATP, and GTP, providing energy and cofactors for cell proliferation and survival [61]. Previous research has demonstrated that purine metabolites may serve as biomarkers for regulating epithelial cell regeneration and influencing cellular energy status [62, 63]. In this experiment, SARA-related factors damage YRECs by altering purine nucleotide content and significantly affecting enzymes involved in purine metabolism. Additionally, pyrimidine metabolism, another aspect of nucleotide metabolism, was found to be associated with the biological effects of acidosis. Additionally, purines serve as structural components of coenzymes and regulate signal transduction and energy metabolism [64]. Under normal physiologic conditions, the activity of purine metabolism-related enzymes is tightly controlled, maintaining a balanced ratio between purine synthesis and purine degradation in the cell [65]. Research examining the global gene expression profiles of sodium butyrate-regulated bovine kidney epithelial cells found that abnormal nucleotide metabolism causes cell cycle arrest, which affects cell proliferation and growth [66]. Furthermore, nucleotides not only serve as substrates for absorption but also influence epithelial cell differentiation, as the proliferation of gastrointestinal epithelial cells relies on nucleotide uptake and metabolism [67].

The main amino acid metabolic pathways affected by the etiology of SARA-related treatments were arginine biosynthesis and glutathione, proline and arginine metabolism, glutamate, aspartate, and alanine metabolism, aminoacyl-tRNA biosynthesis, and beta-alanine metabolism. The semi-essential amino acid arginine is implicated in multiple host cell functions, such as the regulation of cell proliferation, immune response, oxidative stress, and apoptosis [68, 69]. The tight regulation of arginine metabolism is modulated by factors such as diet, cytokines, and hormones [70]. At the same time, studies have discovered an association between aberrant arginine metabolism and the onset and progression of a variety of diseases, such as cancer and infections [71, 72]. In this study, amino acids such as L-arginine, L-ornithine, spermidine, and L-lysine showed lower concentrations compared to the control group, indicating the ability of high-concentration SCFA to inhibit cell growth. Glutathione, an essential component of the cellular oxidative defense system, can increase intracellular NADPH levels to protect cells from peroxide and free radical damage [73, 74]. Numerous studies have demonstrated glutathione depletion triggers autophagy, ferroptosis, and premature cellular senescence in epithelial cells [73, 75, 76]. The approximately 5-fold increase in oxidized l-glutathione concentration at high SCFA concentrations indicated oxidative damage to the cells. Similarly, low pH also induced oxidative damage to YRECs. Early studies have confirmed that SCFA can regulate metabolic, inflammatory, and neural pathways, affecting host metabolism and maintaining energy homeostasis through various complementary mechanisms [77]. In general, cellular metabolic disorders further lead to toxic damage, in which purine metabolism abnormalities may play a key role. Purine nucleotides synthesized and degraded in purine metabolism play a key role in DNA and RNA synthesis and repair within the cell nucleus, essential for maintaining the functionality and stability of the nucleus [78]. In our experimental results, many of the identified proteins that exhibited quantitative differences are located within the nucleus. The disturbances in amino acid metabolism contribute to oxidative stress in cells, decreased energy, and the suppression of RNA and DNA synthesis [79].

The rumen epithelium is characterized by high energy demand and a high concentration of mitochondria [25]. Mitochondrial energy metabolism is crucial for providing the energy required for cellular processes such as proliferation, transport, contraction, and protection. Mitochondria generate approximately 90% of the energy needed for cellular activities via oxidative phosphorylation (OXPHOS) which oxidizes substrates from the TCA to produce ATP [80]. In the present study, high concentration of SCFA and low pH acidic environment significantly inhibited the OXPHOS pathway, causing mitochondrial dysfunction and impairing cellular processes. Key components involved in oxidative phosphorylation, including NADH ubiquinone oxidoreductase (complex I, III), ATP synthase, cytochrome c oxidase, and cytochrome b also were inhibited. Previous studies have shown that a high-starch diet increases oxidative stress and phosphorylation, as SCFA is catabolized within ruminal epithelial cells via oxidative pathways, generating ROS in the ruminal epithelium [81, 82]. Therefore, these findings suggest that the accumulation of SCFA and the low pH acidic environment, exceeding the absorption capacity of YRECs, induce cell damage by disrupting mitochondrial oxidative phosphorylation. Apart from its role in energy metabolism, mitochondria are involved in calcium homeostasis, fatty acid β-oxidation, control of the cell cycle signaling, regulation of cell death, and cellular differentiation [83]. Many studies have linked mitochondrial dysfunction to various diseases, including inflammatory bowel disease, aging, and neurodegenerative disorders [84, 85].

Extensive genetic and pharmacological investigations have illustrated the critical regulatory function of the actin cytoskeleton in the assembly of epithelial barrier AJs/TJs and the remodeling of cell junctions under different stress conditions [86]. Among them, the status of epithelial cell-cell junctions is determined by the dynamics of actin-binding proteins, actin motors, and actin filaments. Dynamic rearrangements of the actin cytoskeleton are essential for eukaryotic cell migration, mechanical integrity, cell structure, polarity, and transcriptional modulation [87].

In this experiment, high concentration of SCFA disrupted the filament proteins, integrins, actin-related protein complexes, and myosin in the focal adhesion, modulation of the actin cytoskeleton, and tight junction pathways of epithelial cells, leading to increased epithelial barrier dysfunction. Disruption of the cytoskeleton and various cell junctions also results in increased paracellular permeability, which facilitates the invasion of harmful substances and their rapid systemic dissemination. Accumulating evidence suggests that actin cytoskeleton-related dynamic rearrangement and assembly/disassembly are required for many cellular processes, and approximately 50% of ATP produced by cellular energy metabolism is necessary to support actin cytoskeleton rearrangement, strengthening cell-cell junctions and forming epithelial barriers [88, 89]. Therefore, The integrity of epithelial barriers is easily disrupted by ATP depletion, which also causes the disassembly of peri junctional actin filaments [90]. Our experimental evidence suggests that the disruption of mitochondrial metabolism caused by high concentration of SCFA, low pH acidic environment, and LPS indirectly affects cytoskeletal exacerbating impairment of nutrient absorption through both transcellular and paracellular pathways.

In the signal pathway regulating cytotoxic damage, high concentration of SCFA mainly affect the Wnt signaling pathway, p53 signaling pathway, PI3K-Akt signaling pathway, and AMPK signaling pathway. In recent decades, an increasing number of studies have identified and characterized various cellular processes regulated by the Wnt signaling pathway, including cell survival and differentiation, tumorigenesis, immune microenvironment regulation, and stem cell proliferation [91]. Specifically, active Wnt signaling is necessary to ensure epithelial homeostasis in the intestines, and pathway suppression causes crypt loss and tissue degradation [92]. In this experiment, the Wnt signaling pathway was significantly inhibited during the epithelial cell homeostasis destruction process, which indirectly indicated that this signaling pathway performed a vital part in the toxic damage of SCFA on YRECs. The PI3K-Akt signaling pathway significantly affects the signal pathway of low pH acidic environment regulating epithelial cell function. Most studies show that PI3K/AKT/mTOR signal pathway was the main target of cytoskeleton-dependent control, which significantly affects cytoskeleton changes and mediates many key cell functions (cell growth, survival, metabolism, exercise, etc.) [93]. The potential molecular mechanism of LPS-induced toxicity of YRECs may involve the intracellular AMPK, p53, NF kappa B signaling pathways, and Toll-like receptors (TLRs). LPS binds to the TLR, intensifies (NF-κB/AMPK signaling expression, and interacts with ruminal epithelial cells, triggering an immune response, oxidative stress, energy metabolism disturbance, and mitochondrial damage [94].

In comparison with previous research findings, our study further elucidates the cellular-level details of the pathogenesis of SARA, offering potential therapeutic targets and biomarkers. However, it is important to note some limitations of this study. Firstly, our experimental design primarily focused on in vitro cell models and has not been validated in animal models or clinical trials. In isolated and cultured YRECs, the basal layer cells are primarily active, facilitating investigations into the effects of distinct rumen internal environments on various aspects of YRECs [30]. The mechanisms underlying rumen tissue damage in pathogenic factors of SARA involve a combination of epithelial barrier disruption, inflammation, oxidative stress, microbial dysbiosis, metabolic disturbances, and cellular damage processes like apoptosis and necrosis [55]. Additionally, due to limitations in experimental conditions, we only investigated the effects of a few key factors on cells, while many other potential factors influencing SARA await further investigation. Therefore, future research should continue to explore the pathogenesis of SARA and validate our experimental results to facilitate further advancements in this field.

## Conclusions

**Fig. 8:**
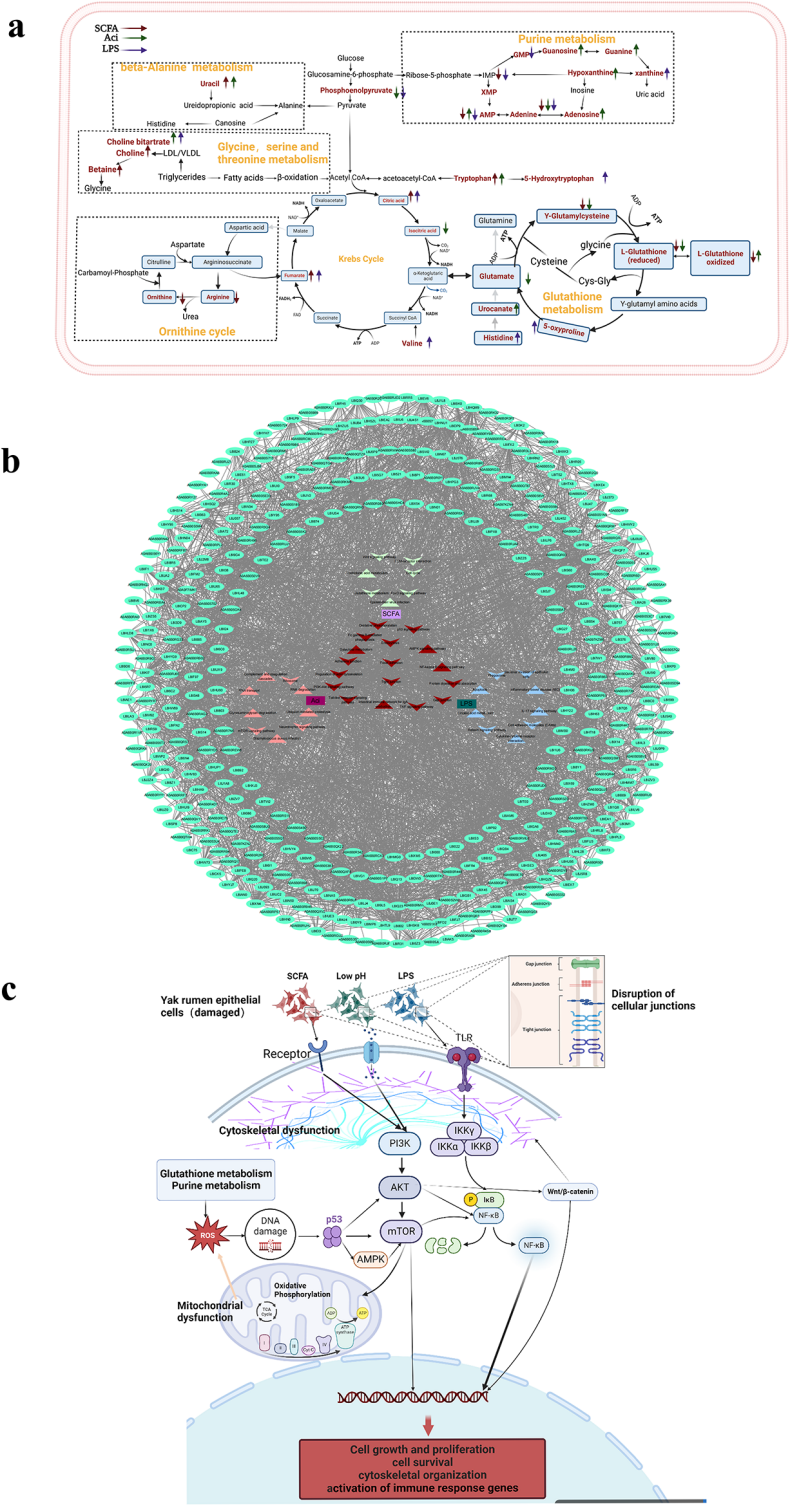
Overview of the main proteomic and metabolomic changes induced by SCFA, Aci, and LPS factors related to ruminal acidosis respectively. (**a**) Integrated metabolic network pathways of metabolic disorders caused by factors related to SARA in YRECs. Different arrow colors represent different factors associated with SARA, with upward arrows representing upregulated expression and downward arrows representing down-regulated expression. The metabolites highlighted in red within the enriched metabolic signaling pathways affected by different treatment groups in YRECs are all significantly different (*P* < 0.05). (**b**) Interaction network of differentially expressed proteins in major changed pathways exposed by SCFA, Aci, and LPS. The green ovals on the periphery of the network represent proteins with significantly differential expression in the enriched signaling pathways (*P* < 0.05). (**c**) Proposed signaling and metabolic pathways for SCFA, Aci, and LPS-induced epithelial toxic injury in YRECs

In this study, using in vitro cultured YRECs as a model, we conducted a comprehensive assessment and systematically investigated the significant effects of SCFA, low pH acidic environments, or LPS associated with SARA etiology on cell proliferation, protein synthesis, and metabolism, elucidating the underlying regulatory mechanisms of cytotoxic damage. SCFA treatment led to floating dead cells, cell shrinkage, and widened intercellular gaps, while low pH disrupted the morphology of epithelial cells, and the impact of LPS on cell viability exhibited a concentration-dependent effect. Furthermore, metabolomic and proteomic analyses revealed significant effects of SCFA, low pH, and LPS treatments on cellular metabolism and protein expression, involving multiple metabolic pathways and signaling pathways. Further analysis revealed that high concentration of SCFA resulted in differential expression of multiple proteins in YRECs, affecting cellular processes such as cytoskeleton organization, tight junction, cell cycle, oxidative phosphorylation, and cell signaling, including Wnt, TGF-beta, Notch signaling pathway. Similarly, low pH treatment resulted in differential protein expression, primarily affecting extracellular matrix, immune regulation, regulation of actin cytoskeleton, tight junction, gap junction metabolic pathways and protein signaling pathway, such as mTOR, Toll-like receptor, TNF signaling pathway. LPS treatment also induced differential protein expression in various signaling pathways, such as TNF, IL-17, Toll-like receptor, and NF-κB, particularly those related to inflammatory responses and immune regulation. Integrated analysis of metabolomic and proteomic data demonstrated alterations in multiple metabolic pathways and protein functions in response to SCFA, low pH, and LPS treatments, including nucleotide metabolism, oxidative phosphorylation, cell cycle regulation, and inflammatory responses. Further correlation analysis highlighted the close relationship between metabolites and proteins, emphasizing their interactions in cellular processes (Fig. 8).

Finally, experimental validation confirmed the molecular mechanisms underlying cellular damage induced by SCFA, low pH, and LPS treatments, including disruption of the cell cycle, mitochondrial dysfunction, oxidative stress, and increased inflammatory responses, which collectively affect cell survival and function. In conclusion, this study provides valuable insights into the effects of SCFA, low pH, and LPS on YRECs, elucidating their mechanisms of cellular damage from morphological, metabolomic, and proteomic perspectives. These findings contribute to a better understanding of the pathophysiological processes underlying SARA and lay a foundation for further research in this field. Further studies are needed to fully understand these complex relationships and pathways underlying metabolomic alterations and their cellular effects in the context of SARA.

### Electronic supplementary material

Below is the link to the electronic supplementary material.

Supplementary Material 1

Supplementary Material 2

Supplementary Material 3

Supplementary Material 4

Supplementary Material 5

Supplementary Material 6

## Data Availability

The datasets used and/or analysed during the current study are available from the corresponding author on reasonable request.

## References

[1] Steele MA, Penner GB, Chaucheyras-Durand F (2016). Development and physiology of the rumen and the lower gut: targets for improving gut health. J Dairy Sci.

[2] dquo BARCROFTJ, McAnally R, Phillipson A (1944). Absorption of volatile acids from the alimentary tract of the sheep and other animals. J Exp Biol.

[3] Allen MS (1997). Relationship between fermentation acid production in the rumen and the requirement for physically effective fiber. J Dairy Sci.

[4] Elmhadi ME, Ali DK, Khogali MK, Wang H. Subacute ruminal acidosis in dairy herds: microbiological and nutritional causes, consequences, and prevention strategies. Anim Nutr 2022. 10.1016/j.aninu.2021.12.008 PMC9168481 35702144

[5] Owens F, Secrist D, Hill W, Gill D (1998). Acidosis in cattle: a review. J Anim Sci.

[6] Nagaraja T, Titgemeyer E (2007). Ruminal acidosis in beef cattle: the current microbiological and nutritional outlook. J Dairy Sci.

[7] Kolver E, De Veth M (2002). Prediction of ruminal pH from pasture-based diets. J Dairy Sci.

[8] Meissner S, Hagen F, Deiner C, Günzel D, Greco G, Shen Z, Aschenbach JR (2017). Key role of short-chain fatty acids in epithelial barrier failure during ruminal acidosis. J Dairy Sci.

[9] Plaizier J, Mesgaran MD, Derakhshani H, Golder H, Khafipour E, Kleen J, Lean I, Loor J, Penner G, Zebeli Q (2018). Enhancing gastrointestinal health in dairy cows. Animal.

[10] Baaske L, Gäbel G, Dengler F (2020). Ruminal epithelium: a checkpoint for cattle health. J Dairy Res.

[11] Guo J, Chang G, Zhang K, Xu L, Jin D, Bilal MS, Shen X. Rumen-derived lipopolysaccharide provoked inflammatory injury in the liver of dairy cows fed a high-concentrate diet. Oncotarget 2017, 8(29). 10.18632/oncotarget.18151 PMC5564522 28596485

[12] Oetzel GR (2017). Diagnosis and management of subacute ruminal acidosis in dairy herds. Veterinary Clinics: Food Anim Pract.

[13] Han G, Gao X, Duan J, Zhang H, Zheng Y, He J, Huo N, Pei C, Li H, Gu S (2021). Effects of yeasts on rumen bacterial flora, abnormal metabolites, and blood gas in sheep with induced subacute ruminal acidosis. Anim Feed Sci Technol.

[14] Wiener G. The yak, an essential element of the high altitude regions of Central Asia. *Études mongoles et sibériennes, centrasiatiques et tibétaines* 2013(43–44).

[15] Wang J, Zhang R, Zhang L, Wang C, Shao B, Wang J (2015). Histomorphometric adaptation of Yak (Bos grunniens) Abomasum to the Qinghai-Tibetan Plateau Environment. Int J Morphology.

[16] Liu X, Gao J, Liu S, Cheng Y, Hao L, Liu S, Zhu W (2023). The uniqueness and superiority of energy utilization in yaks compared with cattle in the highlands: a review. Anim Nutr.

[17] Hu C, Ding L, Jiang C, Ma C, Liu B, Li D, Degen AA. Effects of Management, Dietary Intake, and genotype on Rumen morphology, fermentation, and Microbiota, and on meat quality in yaks and cattle. Front Nutr 2021, 8. 10.3389/fnut.2021.755255 PMC8632495 34859030

[18] Zhang X-L, Xu T-W, Wang X-G, Geng Y-Y, Liu H-J, Hu L-Y, Zhao N, Kang S-P, Zhang W-M, Xu S-X (2020). The Effect of transitioning between feeding methods on the Gut Microbiota Dynamics of Yaks on the Qinghai–Tibet Plateau. Animals.

[19] Liu YX, Ma XM, Xiong L, Wu XY, Liang CN, Bao PJ, Yu QL, Yan P (2020). Effects of Intensive Fattening with total mixed rations on Carcass Characteristics, Meat Quality, and Meat Chemical composition of yak and mechanism based on serum and transcriptomic profiles. Front Vet Sci.

[20] Xu C, Liu W, Sun B, Zhang S, Zhang S, Yang Y, Lei Y, Chang L, Xie P, Suo H. Multi-omics Analysis reveals a dependent relationship between Rumen Bacteria and Diet of Grass-and grain-Fed yaks. Front Microbiol 2021:2278. 10.3389/fmicb.2021.642959 PMC8377600 34421832

[21] Jia J, Liang C, Wu X, Xiong L, Bao P, Chen Q, Yan P (2021). Effect of high proportion concentrate dietary on Ashdan Yak jejunal barrier and microbial function in cold season. Res Vet Sci.

[22] Jiang Y, Dai P, Dai Q, Ma J, Wang Z, Hu R, Zou H, Peng Q, Wang L, Xue B (2022). Effects of the higher concentrate ratio on the production performance, ruminal fermentation, and morphological structure in male cattle-yaks. Veterinary Med Sci.

[23] Zhou J, Liu H, Zhong C, Degen A, Yang G, Zhang Y, Qian J, Wang W, Hao L, Qiu Q (2018). Apparent digestibility, rumen fermentation, digestive enzymes and urinary purine derivatives in yaks and Qaidam cattle offered forage-concentrate diets differing in nitrogen concentration. Livest Sci.

[24] Zhang Z, Xu D, Wang L, Hao J, Wang J, Zhou X, Wang W, Qiu Q, Huang X, Zhou J (2016). Convergent evolution of rumen microbiomes in high-altitude mammals. Curr Biol.

[25] Graham C, Simmons NL (2005). Functional organization of the bovine rumen epithelium. AJP Regul Integr Comp Physiol.

[26] Baldwin RL, Connor EE (2017). Rumen function and development. Veterinary Clinics: Food Anim Pract.

[27] Na SW. Understanding the role of rumen epithelial host-microbial interactions in cattle feed efficiency. Anim Nutr 2022. 10.1016/j.aninu.2022.04.002 PMC9117530 35647325

[28] Baldwin VRL. Use of isolated ruminal epithelial cells in the study of rumen metabolism. J Nutr (2):293S. 10.1093/jn/128.2.293S 9478009

[29] Russell C, Rahman A, Mohammed AR (2013). Application of genomics, proteomics and metabolomics in drug discovery, development and clinic. Therapeutic Delivery.

[30] Wang J, Hu R, Wang Z, Guo Y, Wang S, Zou H, Peng Q, Jiang Y. Establishment of Immortalized Yak Ruminal Epithelial Cell Lines by Lentivirus-Mediated SV40T and hTERT Gene Transduction. *Oxidative Medicine and Cellular Longevity* 2022, 2022. 10.1155/2022/8128028 PMC8975702 35368868

[31] Zhan K, Jiang MC, Gong, Xiaoxiao, Zhao GQ. Effect of short-chain fatty acids on the expression of genes involved in short-chain fatty acid transporters and inflammatory response in goat jejunum epithelial cells. Vitro Cell Dev Biology Anim J Tissues Cult Association 2018. 10.1007/s11626-017-0226-2 29532321

[32] Sellick CA, Hansen R, Stephens GM, Goodacre R, Dickson AJ (2011). Metabolite extraction from suspension-cultured mammalian cells for global metabolite profiling. Nat Protoc.

[33] Yuan M, Breitkopf SB, Yang X, Asara JM (2012). A positive/negative ion–switching, targeted mass spectrometry–based metabolomics platform for bodily fluids, cells, and fresh and fixed tissue. Nat Protoc.

[34] Zheng H-H, Du C-T, Zhang Y-Z, Yu C, Huang R-L, Tang X-Y, Xie G-H (2022). Identification of Canine Pyometra-Associated metabolites using untargeted metabolomics. Int J Mol Sci.

[35] Su A, Chen X, Zhang Z, Xu B, Wang C, Xu Z. Integrated transcriptomic and metabolomic analysis of rat serum to investigate potential target of puerarin in the treatment post-traumatic stress disorder. Annals Translational Med 2021, 9(24). 10.21037/atm-21-6009 PMC8756214 35071465

[36] Zhang H, Liu T, Zhang Z, Payne SH, Zhang B, McDermott JE, Zhou J-Y, Petyuk VA, Chen L, Ray D (2016). Integrated proteogenomic characterization of human high-grade serous ovarian cancer. Cell.

[37] Liu J-F, Wu S-F, Liu C, Chen H-Z, Yang J. Integrated transcriptome, proteome, acetylome, and metabolome profiling of mouse liver during normal aging. 2020.

[38] Livak (2001). Analysis of relative gene expression data using real-time quantitative PCR and the 2– ∆∆CT method. Methods.

[39] Wang J, Jin Y, Wu S, Yu H, Zhao Y, Fang H, Shen J, Zhou C, Fu Y, Li R (2019). Deoxynivalenol induces oxidative stress, inflammatory response and apoptosis in bovine mammary epithelial cells. J Anim Physiol Anim Nutr.

[40] Katanaev VL, Wymann MP (1998). Microquantification of Cellular Andin VitroF-Actin by Rhodamine Phalloidin Fluorescence Enhancement. Anal Biochem.

[41] Pendergrass W, Wolf N, Poot M (2004). Efficacy of MitoTracker Green™ and CMXrosamine to measure changes in mitochondrial membrane potentials in living cells and tissues. Cytometry Part A: J Int Soc Anal Cytol.

[42] Wen B, Mei Z, Zeng C, Liu S (2017). metaX: a flexible and comprehensive software for processing metabolomics data. BMC Bioinformatics.

[43] Jones P, Binns D, Chang H-Y, Fraser M, Li W, McAnulla C, McWilliam H, Maslen J, Mitchell A, Nuka G (2014). InterProScan 5: genome-scale protein function classification. Bioinformatics.

[44] Huang DW, Sherman BT, Lempicki RA (2009). Bioinformatics enrichment tools: paths toward the comprehensive functional analysis of large gene lists. Nucleic Acids Res.

[45] Franceschini A, Szklarczyk D, Frankild S, Kuhn M, Simonovic M, Roth A, Lin J, Minguez P, Bork P, Von Mering C (2012). STRING v9. 1: protein-protein interaction networks, with increased coverage and integration. Nucleic Acids Res.

[46] Penner GB. Short chain fatty acid absorption and regulation of ruminal pH. 2019.

[47] Ma J, Shah AM, Wang Z, Fan X (2021). Potential protective effects of thiamine supplementation on the ruminal epithelium damage during subacute ruminal acidosis. Anim Sci J.

[48] Monteiro HF, Faciola AP (2020). Ruminal acidosis, bacterial changes, and lipopolysaccharides. J Anim Sci.

[49] Bugaut M (1987). Occurrence, absorption and metabolism of short chain fatty acids in the digestive tract of mammals. Comp Biochem Physiol Part B: Comp Biochem.

[50] Sakata T, Yajima T (1984). Influence of short chain fatty acids on the epithelial cell division of digestive tract. QJ Exp Physiol.

[51] Kristensen N, Danfaer A, Agergaard N (1998). Absorption and metabolism of short-chain fatty acids in ruminants. Arch Anim Nutr.

[52] Pang K, Dai D, Yang Y, Wang X, Liu S, Huang W, Xue B, Chai S, Wang S. Effects of high concentrate rations on ruminal fermentation and microbiota of yaks. Front Microbiol 2022, 13. 10.3389/fmicb.2022.957152 PMC9558216 36246255

[53] Dijkstra J, Ellis J, Kebreab E, Strathe A, López S, France J, Bannink A (2012). Ruminal pH regulation and nutritional consequences of low pH. Anim Feed Sci Technol.

[54] Gui H, Shen Z (2016). Concentrate diet modulation of ruminal genes involved in cell proliferation and apoptosis is related to combined effects of short-chain fatty acid and pH in rumen of goats. J Dairy Sci.

[55] Aschenbach JR, Zebeli Q, Patra AK, Greco G, Amasheh S, Penner GB. Symposium review: The importance of the ruminal epithelial barrier for a healthy and productive cow. *Journal of Dairy Science* 2019, 102(2):1866–1882. 10.3168/jds.2018-15243 30580938

[56] Kent-Dennis C, Aschenbach JR, Griebel PJ, Penner GB. Effects of lipopolysaccharide exposure in primary bovine ruminal epithelial cells. J Dairy Sci 2020, 103(10). 10.3168/jds.2020-18652 32747102

[57] Hirotani Y, Ikeda K, Kato R, Myotoku M, Umeda T, Ijiri Y, Tanaka K (2008). Protective effects of lactoferrin against intestinal mucosal damage induced by lipopolysaccharide in human intestinal Caco-2 cells. Yakugaku Zasshi.

[58] Nägele T (2014). Linking metabolomics data to underlying metabolic regulation. Front Mol Biosci.

[59] Zhang A, Sun H, Wang P, Han Y, Wang X (2012). Modern analytical techniques in metabolomics analysis. Analyst.

[60] Zhang A-h, Sun H, Han Y, Yan G-l, Yuan Y, Song G-c, Yuan X-x, Xie N, Wang X-j (2013). Ultraperformance liquid chromatography–mass spectrometry based comprehensive metabolomics combined with pattern recognition and network analysis methods for characterization of metabolites and metabolic pathways from biological data sets. Anal Chem.

[61] Moriwaki Y, Yamamoto T, Higashino K (1999). Enzymes involved in purine metabolism-a review of histochemical localization and functional implications. Histol Histopathol.

[62] Wang L, Wang X, Shi Z, Shen L, Zhang J, Zhang J (2021). Bovine milk exosomes attenuate the alteration of purine metabolism and energy status in IEC-6 cells induced by hydrogen peroxide. Food Chem.

[63] Nagahama Y, Shimoda M, Mao G, Singh SK, Kozakai Y, Sun X, Motooka D, Nakamura S, Tanaka H, Satoh T. Regnase-1 controls colon epithelial regeneration via regulation of mTOR and purine metabolism. *Proceedings of the National Academy of Sciences* 2018, 115(43):11036–11041. 10.1073/pnas.1809575115 PMC6205455 30297433

[64] Tang Z, Ye W, Chen H, Kuang X, Guo J, Xiang M, Peng C, Chen X, Liu H (2019). Role of purines in regulation of metabolic reprogramming. Purinergic Signalling.

[65] Szczurek P, Mosiichuk N, Woliński J, Yatsenko T, Grujic D, Lozinska L, Pieszka M, Święch E, Pierzynowski SG, Goncharova K (2017). Oral uricase eliminates blood uric acid in the hyperuricemic pig model. PLoS ONE.

[66] Li C-j, Li RW, Wang Y-h, Elsasser TH (2007). Pathway analysis identifies perturbation of genetic networks induced by butyrate in a bovine kidney epithelial cell line. Funct Integr Genom.

[67] Sanderson IR, He Y (1994). Nucleotide uptake and metabolism by intestinal epithelial cells. J Nutr.

[68] Albaugh VL, Pinzon-Guzman C, Barbul A (2017). Arginine—dual roles as an onconutrient and immunonutrient. J Surg Oncol.

[69] Wu G, Bazer FW, Satterfield MC, Gilbreath KR, Posey EA, Sun Y. L-Arginine nutrition and metabolism in ruminants. Recent advances in Animal Nutrition and Metabolism. Springer; 2022. pp. 177–206. 10.1007/978-3-030-85686-1_10 34807443

[70] Wu G, Morris SM (1998). Arginine metabolism: nitric oxide and beyond. Biochem J.

[71] Al-Koussa H, El Mais N, Maalouf H, Abi-Habib R, El-Sibai M (2020). Arginine deprivation: a potential therapeutic for cancer cell metastasis? A review. Cancer Cell Int.

[72] Zhang H, Peng A, Yu Y, Guo S, Wang M, Wang H (2019). L-arginine protects ovine intestinal epithelial cells from lipopolysaccharide-induced apoptosis through alleviating oxidative stress. J Agric Food Chem.

[73] Sun Y, Zheng Y, Wang C, Liu Y (2018). Glutathione depletion induces ferroptosis, autophagy, and premature cell senescence in retinal pigment epithelial cells. Cell Death Dis.

[74] Wu G, Fang Y-Z, Yang S, Lupton JR, Turner ND (2004). Glutathione metabolism and its implications for health. J Nutr.

[75] White AC, Thannickal VJ, Fanburg BL (1994). Glutathione deficiency in human disease. J Nutr Biochem.

[76] Lash L (2011). Renal membrane transport of glutathione in toxicology and disease. Vet Pathol.

[77] Barrea L, Muscogiuri G, Annunziata G, Laudisio D, Pugliese G, Salzano C, Colao A, Savastano S (2019). From gut microbiota dysfunction to obesity: could short-chain fatty acids stop this dangerous course?. Hormones.

[78] Chitrakar I, Kim-Holzapfel DM, Zhou W, French JB (2017). Higher order structures in purine and pyrimidine metabolism. J Struct Biol.

[79] Zhao X, Abulikemu A, Lv S, Qi Y, Duan J, Zhang J, Chen R, Guo C, Li Y, Sun Z (2021). Oxidative stress-and mitochondrial dysfunction-mediated cytotoxicity by silica nanoparticle in lung epithelial cells from metabolomic perspective. Chemosphere.

[80] Koo MJ, Rooney KT, Choi ME, Ryter SW, Choi AM, Moon J-S (2015). Impaired oxidative phosphorylation regulates necroptosis in human lung epithelial cells. Biochem Biophys Res Commun.

[81] Penner G, Steele M, Aschenbach J, McBride B. Ruminant Nutrition Symposium: Molecular adaptation of ruminal epithelia to highly fermentable diets. *Journal of Animal Science* 2011, 89(4):1108–1119. 10.2527/jas.2010-3378 20971890

[82] Guo Y, Xu X, Zou Y, Yang Z, Li S, Cao Z (2013). Changes in feed intake, nutrient digestion, plasma metabolites, and oxidative stress parameters in dairy cows with subacute ruminal acidosis and its regulation with pelleted beet pulp. J Anim Sci Biotechnol.

[83] Smith RA, Hartley RC, Cocheme HM, Murphy MP (2012). Mitochondrial pharmacology. Trends Pharmacol Sci.

[84] Niyazov DM, Kahler SG, Frye RE (2016). Primary mitochondrial disease and secondary mitochondrial dysfunction: importance of distinction for diagnosis and treatment. Mol Syndromol.

[85] Pieczenik SR, Neustadt J (2007). Mitochondrial dysfunction and molecular pathways of disease. Exp Mol Pathol.

[86] Lechuga S, Ivanov AI (2021). Actin cytoskeleton dynamics during mucosal inflammation: a view from broken epithelial barriers. Curr Opin Physiol.

[87] Blanchoin L, Boujemaa-Paterski R, Sykes C, Plastino J (2014). Actin dynamics, architecture, and mechanics in cell motility. Physiol Rev.

[88] Bernstein BW, Bamburg JR (2003). Actin-ATP hydrolysis is a major energy drain for neurons. J Neurosci.

[89] Bays JL, Campbell HK, Heidema C, Sebbagh M, DeMali KA (2017). Linking E-cadherin mechanotransduction to cell metabolism through force-mediated activation of AMPK. Nat Cell Biol.

[90] JanssenDuijghuijsen LM, Grefte S, De Boer VC, Zeper L, Van Dartel DA, Van der Stelt I, Bekkenkamp-Grovenstein M, van Norren K, Wichers HJ, Keijer J (2017). Mitochondrial ATP depletion disrupts Caco-2 monolayer integrity and internalizes claudin 7. Front Physiol.

[91] Barker N. The canonical Wnt/β-catenin signalling pathway. Wnt Signal 2008:5–15. 10.1007/978-1-59745-249-6_1 19099242

[92] Kuhnert F, Davis CR, Wang H-T, Chu P, Lee M, Yuan J, Nusse R, Kuo CJ. Essential requirement for Wnt signaling in proliferation of adult small intestine and colon revealed by adenoviral expression of Dickkopf-1. *Proceedings of the National Academy of Sciences* 2004, 101(1):266–271. 10.1073/pnas.2536800100 PMC314174 14695885

[93] Deng S, Leong HC, Datta A, Gopal V, Kumar AP, Yap CT (2022). PI3K/AKT Signaling Tips the Balance of Cytoskeletal forces for Cancer Progression. Cancers.

[94] Ma Y, Elmhadi M, Wang C, Li Z, Zhang H, He B, Zhao X, Zhang Z, Wang H. Thiamine Supplementation Alleviates Lipopolysaccharide-Triggered Adaptive Inflammatory Response and Modulates Energy State via Suppression of NFκB/p38 MAPK/AMPK Signaling in Rumen Epithelial Cells of Goats. *Antioxidants* 2022, 11(10):2048. 10.3390/antiox11102048 PMC9598694 36290775

